# Digital control of wide gamut color spun yarns enabled by four primary grid based mixing model

**DOI:** 10.1038/s41598-025-19972-x

**Published:** 2025-10-15

**Authors:** Peng Cui, Yuan Xue, Yuchen Liu

**Affiliations:** 1 Fujian Provincial Engineering Technology Research Center of Industrial Design and Intelligent Manufacturing, Liming Vocational University, No. 298 West Tonggang Street, Quanzhou, 362000 Fujian China; 2 School of New Materials and Shoes & Clothing Engineering, Liming Vocational University, No. 298 West Tonggang Street, Quanzhou, 362000 Fujian China; 3 Footwear and Apparel Product Structural Innovation and Functional R&D Collaborative Innovation Center, Liming Vocational University, Quanzhou, 362000 Fujian China; 4 School of Textile Science and Engineering, Jiangnan University, No.1800 Lihu Ave., Wuxi, 214122 Jiangsu China

**Keywords:** Color-spun yarns, Wide-gamut color mixing, Digital textile manufacturing, Three-channel numerical control spinning, Engineering, Materials science

## Abstract

This paper introduces a digital control system for producing wide-gamut color-spun yarns by integrating a four-primary (Cyan, Magenta, Yellow, Grey) grid-based mixing model with a three-channel computer numerical control (CNC) rotor spinning system. The framework was validated by producing and testing 90 systematically varied yarn samples. The resulting yarns demonstrated precise, predictable colors, with exceptional agreement between the predicted model and measured fabric colors across the entire gamut. Furthermore, comprehensive testing confirmed that the yarns’ mechanical properties, evenness, and hairiness meet or exceed industry standards. This research provides a systematic, mathematically rigorous approach that transforms color-spun yarn production from a craft-based process into a precision-controlled digital manufacturing technology.

**Supplementary Information:**

The online version contains supplementary material available at 10.1038/s41598-025-19972-x.

## Introduction

Precise color control represents one of the most significant challenges in modern textile manufacturing, particularly for products where aesthetic value is paramount^1–4^. The increasing demand for textiles with complex colorations, coupled with growing environmental concerns, has driven innovation in sustainable coloration methods^5–7^. Color-spun yarn production—where fibers are dyed prior to spinning rather than afterward—has emerged as a transformative approach that simultaneously addresses environmental constraints and market demands for customization. This “first-color, then-spin" methodology reduces wastewater pollution by approximately 50% while enabling unprecedented flexibility for small-batch, multi-variety production that responds to rapidly changing market preferences^6–9^. With over 10 million spindles currently dedicated to color-spun yarn production in China alone, the economic and environmental impact of this approach is substantial.

Despite these considerable advantages, achieving accurate colorimetric prediction and consistent reproduction in color-spun yarns remains exceptionally challenging^10,11^. This difficulty stems from several interrelated factors, creating a highly non-linear relationship between fiber mixture ratios and perceived color. Different fiber types exhibit varying optical properties including light absorption, scattering, and reflectance characteristics that significantly affect color perception^12,13^. Processing methods introduce additional complexity through non-uniform mixing of differently colored fibers within the yarn structure, creating micro-scale color variations that affect overall appearance^14,15^. Furthermore, environmental lighting conditions significantly affect the perceived color of both yarns and their resultant fabrics, introducing another layer of variability^16^. These multidimensional interactions create a profoundly non-linear relationship between fiber mixture ratios and perceived color that conventional color prediction models struggle to accurately represent.

The textile industry has traditionally relied on experience-based approaches to color-spun yarn production, involving extensive trial-and-error sampling before achieving desired colors^7,17–20^. This resource-intensive process not only limits production efficiency but also constrains the achievable color gamut and reproducibility precision. Moreover, the inability to digitally control color parameters during yarn formation itself has restricted the application of color-spun technology in advanced manufacturing contexts where precision and repeatability are essential requirements^21–23^. While several research initiatives have begun addressing these limitations, existing approaches remain fragmented. Some studies have focused on understanding the mechanisms of color transfer between dyed fibers and resultant yarns^14,24^, while others have explored optimization of blending methods and spinning processes to enhance color consistency^25,26^. Particularly promising are recent innovations involving digital control systems for color fiber blending during the spinning process^27,28^. However, these studies have typically been limited to narrow color ranges or specific fiber types, lacking a comprehensive framework for wide-gamut color prediction and control that would enable true digital manufacturing capabilities.

Recent advances in multi-roving yarn production have demonstrated the critical importance of roving positioning and feeding geometry on resultant yarn properties and color uniformity. Multi-channel spinning systems, particularly three-roving configurations, have emerged as promising approaches for creating complex yarn structures with enhanced functionality^28–30^. Studies by Demir^31^ demonstrated that roving distances significantly affect fiber positioning, packing density, and mechanical properties, with symmetric arrangements enhancing strength and compactness, while asymmetric ones reduce hairiness and improve elongation. No impact on unevenness. Similarly, Cui et al.^32^ found that optimizing roving distance (e.g., symmetric for strength, asymmetric for reduced hairiness) can tailor mechanical properties for applications like high-performance textiles. Chen et al.^33^ examine strand spacing, or roving distance, as a critical factor in filament spread composite-structural yarn (FS-CSY) production, varying it from 1 to 7 mm. The optimal spacing of 3 mm yields peak breaking tenacity (12.93 cN/tex), a 12.26% increase in extension rate compared to 1 mm, better evenness (11.87% at 1 mm), and reduced hairiness (S3 6.31% lower at 1 mm). Wider spacings shift the structure from wrapping to interlocking, increasing perimeter and reducing coverage. Fuzzy evaluation confirms 3 mm as ideal for self-reinforcement, guiding the optimization of composite yarns for enhanced stability and reduced hairiness. Sun et al.^34^ further demonstrated that 3 mm strand spacing optimizes strength and hairiness reduction via symmetric triangle geometry and better fiber control, while 2 mm excels for abrasion. Theoretical models confirm position effects on tension and torque. These findings highlight the critical need for precise geometric control in multi-roving systems. In the context of color-spun yarn production, roving positioning becomes even more critical due to its direct impact on color mixing uniformity. Studies by Liu et al.^7^ showed that in section-color yarn spinning, the white roving is fed continuously into the middle roller, while the color roving is fed discontinuously into the back roller, and “feeding deviation” (slippage of the separation point between rovings due to velocity differences) causes uneven blending, resulting in slubs and thin places that affect color uniformity and distribution. Zhang et al.^35^ explores qualities of colored fancy yarns from 60/40 polyester/cotton blends, comparing colored AB Sirospun, multicolored, and section-color types spun on an EJM128K frame after reactive dyeing, which increased fiber thickness but reduced strength. Testing revealed AB Sirospun yarn excelled in breaking strength (290.58 cN), evenness (coefficient of variation of yarn mass (CVm) 14.02%), and hairiness (H 2.69), outperforming multicolored (poorest) and section-color due to its twisting mechanism, with all achieving high-class fastness. These studies underscore the importance of precise mechanical control in achieving predictable color outcomes in multi-roving systems. However, existing research has predominantly focused on conventional spinning approaches with limited digital control capabilities. Most studies employ fixed roving positions and rely on pre-mixing strategies rather than real-time adjustment of individual roving contributions. Furthermore, the majority of research has been conducted on a narrow color range or specific fiber combinations, limiting the generalizability of findings to wide-gamut color production. The integration of digital control systems with multi-roving spinning, particularly for achieving wide-color gamut control, remains largely unexplored in the literature, representing a significant gap that our research aims to address.

To address these fundamental limitations, an integrated approach is proposed that combines theoretical color modeling with advanced manufacturing technology. Our research establishes a wide-color gamut mixing model based on four primary colored fibers (Magenta, Yellow, Cyan, and Grey), constructing a systematic grid-based framework with 12.5% discrete increments that enables precise control over hue, saturation, and lightness. By implementing this model through a three-channel computer numerical control (CNC) rotor spinning system^27,28^, we establish a three-factor control mechanism linking channel draft ratios, colored fiber blending proportions, and resultant yarn colors. This bidirectional approach represents a significant advancement in that it enables both predictive capabilities (determining yarn color from known fiber blend ratios) and inverse design capabilities (determining necessary blend ratios to achieve target colors)—a transformative capability that enhances both design flexibility and manufacturing efficiency while eliminating extensive sampling requirements.

The significance of our approach lies in its ability to achieve real-time digital control over color parameters during yarn formation itself, effectively digitizing the entire color creation process. This integration of color theory with precision manufacturing enables unprecedented control across the wide color gamut while maintaining the environmental benefits of the color-spun approach. To validate the theoretical framework, three distinct series of colored yarns were produced and tested: those with equal lightness but different hues, equal hue but different lightness levels, and equal hue but different saturation levels. Comprehensive testing of these experimental yarns’ evenness, hairiness, and mechanical properties demonstrated that our digitally controlled color-spun yarns not only achieved precise, predictable color outcomes but also maintained physical properties meeting or exceeding industry standards for first-grade products. This research addresses a critical gap in textile manufacturing technology by providing a systematic, mathematically rigorous approach to color prediction and control in color-spun yarns. By establishing the relationships between yarn color values, primary fiber mixing ratios, channel draft ratios, and color space coordinates, we provide both theoretical foundations and practical methodologies for advanced digital control in sustainable textile coloration. Our grid-based model creates a standardized framework that can be readily implemented in manufacturing environments, potentially transforming color-spun yarn production from a craft-based process to a precision-controlled digital manufacturing technology.

## Theoretical and experimental methods

Building upon our previous foundational work in digital color control systems^14,27,28^, this research significantly advances the methodology through comprehensive expansion of technical details and systematic comparative analysis. While our earlier work demonstrated the basic feasibility of computer numerically controlled spinning for color blended yarns^14^, the current study extends this approach by implementing a sophisticated four-primary grid-based mixing model with 12.5% discrete increments across the entire color gamut. Compared to conventional single-channel approaches that rely on pre-blended materials, our three-channel system enables real-time adjustment of individual roving contributions through independent speed control. This approach addresses fundamental spinning challenges through advanced fiber integration mechanisms that ensure uniform fiber distribution throughout the yarn cross-section, effectively preventing color segregation or clustering that could compromise color prediction accuracy. The enhanced roving feeding system design, featuring nested coaxial roller structures with standardized spacing, ensures controlled mixing uniformity during the spinning process. This advancement represents a significant departure from traditional experience-based color-spun yarn production by establishing advanced digital control capabilities that enhance both design flexibility and manufacturing precision. These methodological enhancements demonstrate that our approach successfully bridges the gap between theoretical color modeling and practical manufacturing implementation.

### Design and construction of the wide-gamut color mixing system

To develop a comprehensive and systematically structured dataset for color prediction modeling, a wide-color gamut mixing framework was designed based on four carefully selected primary-colored fibers. These included Grey as the achromatic base, along with three chromatic primaries: Cyan, Magenta, and Yellow. This strategic combination enabled the systematic generation of a broad spectrum of colors with diverse hues, saturations, and lightness levels. To ensure the model’s generalizability, our theoretical framework is developed using the standard subtractive primary colors: Cyan (α), Magenta (β), and Yellow (γ), along with an achromatic Grey (o). In the subsequent experimental sections, this abstract model will be validated using commercially available dyed fibers, which are designated as Red, Yellow, and Blue, to test the framework’s applicability in a practical, industrial context.

We first selected optimal dyes and optimized dyeing processes to obtain three chromatic fibers (α, β, γ) with maximum color purity for use as the three base colors. These were combined with an achromatic fiber (o) to create our four primary system. The weights of these fibers were designated as W_α_, W_β_, W_γ_, and W_o_, with color values represented as C_α_(R_α_,G_α_,B_α_), C_β_(R_β_,G_β_,B_β_), C_γ_(R_γ_,G_γ_,B_γ_), and C_o_(R_o_,G_o_,B_o_). The weights of the four primary fibers were discretized using a 12.5% gradient increment, resulting in the following expression for each discretized fiber weight:

1 $${\text{W}}_{\alpha } = {\text{W}}_{\alpha } \times \frac{{{\text{j}} - 1}}{8}$$

2 $${\text{W}}_{\upbeta }={\text{W}}_{\upbeta }\times \frac{\text{j}-1}{8}$$

3 $${\text{W}}_{\upgamma }={\text{W}}_{\upgamma }\times \frac{\text{j}-1}{8}$$

4 $${\text{W}}_{\text{o}}={\text{W}}_{\text{o}}\times \frac{\text{j}-1}{8}$$

where j = 1, 2, 3, …, 8, 9.

The combination mixing modes for these four primary fibers are expressed as:

5 $${\text{W}}_{{{\text{o}}\alpha \beta }} \left( {{\text{j}}_{1} ,{\text{j}}_{2} ,{\text{j}}_{3} } \right) = {\text{W}}_{{\text{o}}} \left( {{\text{j}}_{1} } \right) + {\text{W}}_{\alpha } \left( {{\text{j}}_{2} } \right) + {\text{W}}_{\beta } \left( {{\text{j}}_{3} } \right)$$

where j_1_, j_2_, j_3_ = 1, 2, 3, …, 8, 9.

The dual-coupling color mixing approach combines two or more colored fibers using discrete weights. We first established equal baseline weights for each primary fiber and applied identical discretization gradients. We then selected portions from each discretized primary fiber weight sequence and combined them to produce systematic series of blended samples. When only combinations that maintain equal total weight to the baseline weight are selected, this mixing approach is termed coupling color mixing. For three primary fibers, we first discretized them according to equal baseline weights and discretization gradients. We then selected two primary fibers for first-level coupling mixing, followed by coupling with the third primary fiber for second-level coupling mixing. Throughout this process, the total weight of each mixed sample remained equal to the baseline weight, with primary fiber mixing ratios varying within the 0% to 100% range. This approach is termed dual-coupling color mixing.

Through the approach described above, we obtained four primary colors based on the four original fibers, using 12.5% as the discrete gradient to control hue, lightness, and saturation. Selecting (W_o_(j_1_)-W_ɑ_(j_2_)-W_β_(j_3_)), (W_o_(j_1_)-W_β_(j_2_)-W_γ_(j_3_)), and (W_o_(j_1_)-W_γ_(j_2_)-W_ɑ_(j_3_)) for ternary combination mixing, the weights of each subsample (W_oβα_(j_1_,j_2_,j_3_), W_oγ_β(j_1_,j_2_,j_3_), W_oαγ_(j_1_,j_2_,j_3_)) can be expressed as:

6 $$\left\{ {\begin{array}{*{20}c} {{\text{W}}_{{{\text{o}}\alpha \beta }} ({\text{j}}_{1} ,{\text{j}}_{2} ,{\text{j}}_{3} ) = \frac{{9 - {\text{j}}_{1} }}{8} \times {\text{W}}_{{\text{o}}} + \frac{{{\text{j}}_{1} - 1}}{8} \times \left( {\frac{{{\text{j}}_{2} - 1}}{8} \times {\text{W}}_{\beta } + \frac{{{\text{j}}_{3} - 1}}{8} \times {\text{W}}_{\alpha } } \right)} \\ {{\text{W}}_{{{\text{o}}\gamma \beta }} ({\text{j}}_{1} ,{\text{j}}_{2} ,{\text{j}}_{3} ) = \frac{{9 - {\text{j}}_{1} }}{8} \times {\text{W}}_{{\text{o}}} + \frac{{{\text{j}}_{1} - 1}}{8} \times \left( {\frac{{{\text{j}}_{2} - 1}}{8} \times {\text{W}}_{\gamma } + \frac{{{\text{j}}_{3} - 1}}{8} \times {\text{W}}_{\beta } } \right)} \\ {{\text{W}}_{{{\text{o}}\alpha \gamma }} ({\text{j}}_{1} ,{\text{j}}_{2} ,{\text{j}}_{3} ) = \frac{{9 - {\text{j}}_{1} }}{8} \times {\text{W}}_{{\text{o}}} + \frac{{{\text{j}}_{1} - 1}}{8} \times \left( {\frac{{{\text{j}}_{2} - 1}}{8} \times {\text{W}}_{\alpha } + \frac{{{\text{j}}_{3} - 1}}{8} \times {\text{W}}_{\gamma } } \right)} \\ \end{array} } \right.$$

where j_1_, j_2_, j_3_ = 1, 2, 3, …, 8, 9

Based on the definition of dual-coupling color mixing, in Eq. (6), we use j_2_ + j_3_ = 10 to keep the combined weight of W_α_ + W_β_ constant, forming the first-level coupling mixing. This is then coupled with Wo to form the second-level coupling mixing, thereby constituting the ternary dual-coupling color mixing. Assuming W_α_ = W_β_ = W_γ_ = W_o_ = W, where W = W_oαβ_(j_1_,j_2_,j_3_) = W_oβγ_(j_1_,j_2_,j_3_) = W_oγα_(j_1_,j_2_,j_3_), and substituting j_3_ = 10—j_2_ into Eq. (3), we derive the weights for each subsample in the ternary dual-coupling color mixing:

7 $$\left\{ {\begin{array}{*{20}c} {{\text{W}}_{{{\text{o}}\alpha \beta }} ({\text{j}}_{1} ,{\text{j}}_{2} ) = \frac{{9 - {\text{j}}_{1} }}{8} \times {\text{W}}_{{\text{o}}} + \frac{{9{\text{j}}_{1} - 9 - {\text{j}}_{1} {\text{j}}_{2} + {\text{j}}_{2} }}{64} \times {\text{W}}_{\alpha } + \frac{{{\text{j}}_{1} {\text{j}}_{2} - {\text{j}}_{1} - {\text{j}}_{2} + 1}}{64} \times {\text{W}}_{\beta } } \\ {{\text{W}}_{{{\text{o}}\beta \gamma }} ({\text{j}}_{1} ,{\text{j}}_{2} ) = \frac{{9 - {\text{j}}_{1} }}{8} \times {\text{W}}_{{\text{o}}} + \frac{{9{\text{j}}_{1} - 9 - {\text{j}}_{1} {\text{j}}_{2} + {\text{j}}_{2} }}{64} \times {\text{W}}_{\beta } + \frac{{{\text{j}}_{1} {\text{j}}_{2} - {\text{j}}_{1} - {\text{j}}_{2} + 1}}{64} \times {\text{W}}_{\gamma } } \\ {{\text{W}}_{{{\text{o}}\gamma \alpha }} ({\text{j}}_{1} ,{\text{j}}_{2} ) = \frac{{9 - {\text{j}}_{1} }}{8} \times {\text{W}}_{{\text{o}}} + \frac{{9{\text{j}}_{1} - 9 - {\text{j}}_{1} {\text{j}}_{2} + {\text{j}}_{2} }}{64} \times {\text{W}}_{\gamma } + \frac{{{\text{j}}_{1} {\text{j}}_{2} - {\text{j}}_{1} - {\text{j}}_{2} + 1}}{64} \times {\text{W}}_{\alpha } } \\ \end{array} } \right.$$

where j_1_, j_2_ = 1, 2, 3, …, 8, 9

Let the mixing ratios of primary fibers x, y, z in the mixed subsample be λ_x_(j_1_,j_2_), λ_y_(j_1_,j_2_), λ_z_(j_1_,j_2_). For W_oαβ_(j_1_,j_2_), the primary fibers in the mixed subsample are x = α, y = β, z = o. For W_oβγ_(j_1_,j_2_), the primary fibers in the mixed subsample are x = β, y = γ, z = o. For W_oγα_(j_1_,j_2_), the primary fibers in the mixed subsample are x = γ, y = α, z = o. From Eq. (4), the mixing ratio of primary fibers in the mixed subsample can be derived as:

8 $$\left\{\begin{array}{c}{\uplambda }_{\text{x}}({\text{j}}_{1},{\text{j}}_{2})=\frac{9{\text{j}}_{1}-{\text{j}}_{1}{\text{j}}_{2}+{\text{j}}_{2}-9}{64}\\ {\uplambda }_{\text{y}}({\text{j}}_{1},{\text{j}}_{2})=\frac{{\text{j}}_{1}{\text{j}}_{2}-{\text{j}}_{1}-{\text{j}}_{2}+1}{64}\\ {\uplambda }_{\text{o}}({\text{j}}_{1},{\text{j}}_{2})=\frac{9-{\text{j}}_{1}}{8}\end{array}\right.$$

where j_1_, j_2_ = 1, 2, 3, …, 9.

Let the color of mixed sample be C(j_1_,j_2_) = [C_r_(j_1_,j_2_), C_g_(j_1_,j_2_), C_b_(j_1_,j_2_)]ᵀ, then:

9 $$\text{C}({\text{j}}_{1},{\text{j}}_{2})=\left[\begin{array}{c}{\text{C}}_{\text{r}}({\text{j}}_{1},{\text{j}}_{2})\\ {\text{C}}_{\text{g}}({\text{j}}_{1},{\text{j}}_{2})\\ {\text{C}}_{\text{b}}({\text{j}}_{1},{\text{j}}_{2})\end{array}\right]=\left[\begin{array}{ccc}{\text{R}}_{\text{x}}& {\text{R}}_{\text{y}}& {\text{R}}_{\text{o}}\\ {\text{G}}_{\text{x}}& {\text{G}}_{\text{y}}& {\text{G}}_{\text{o}}\\ {\text{B}}_{\text{x}}& {\text{B}}_{\text{y}}& {\text{B}}_{\text{o}}\end{array}\right]\left[\begin{array}{c}{\uplambda }_{\text{x}}({\text{j}}_{1},{\text{j}}_{2})\\ {\uplambda }_{\text{y}}({\text{j}}_{1},{\text{j}}_{2})\\ {\uplambda }_{\text{o}}({\text{j}}_{1},{\text{j}}_{2})\end{array}\right]$$

where j_1_, j_2_ = 1, 2, 3, …, 9. In this equation, j_1_ reflects the saturation variation in the color mixing process, while j_2_ reflects the hue variation.

**Fig. 1:**
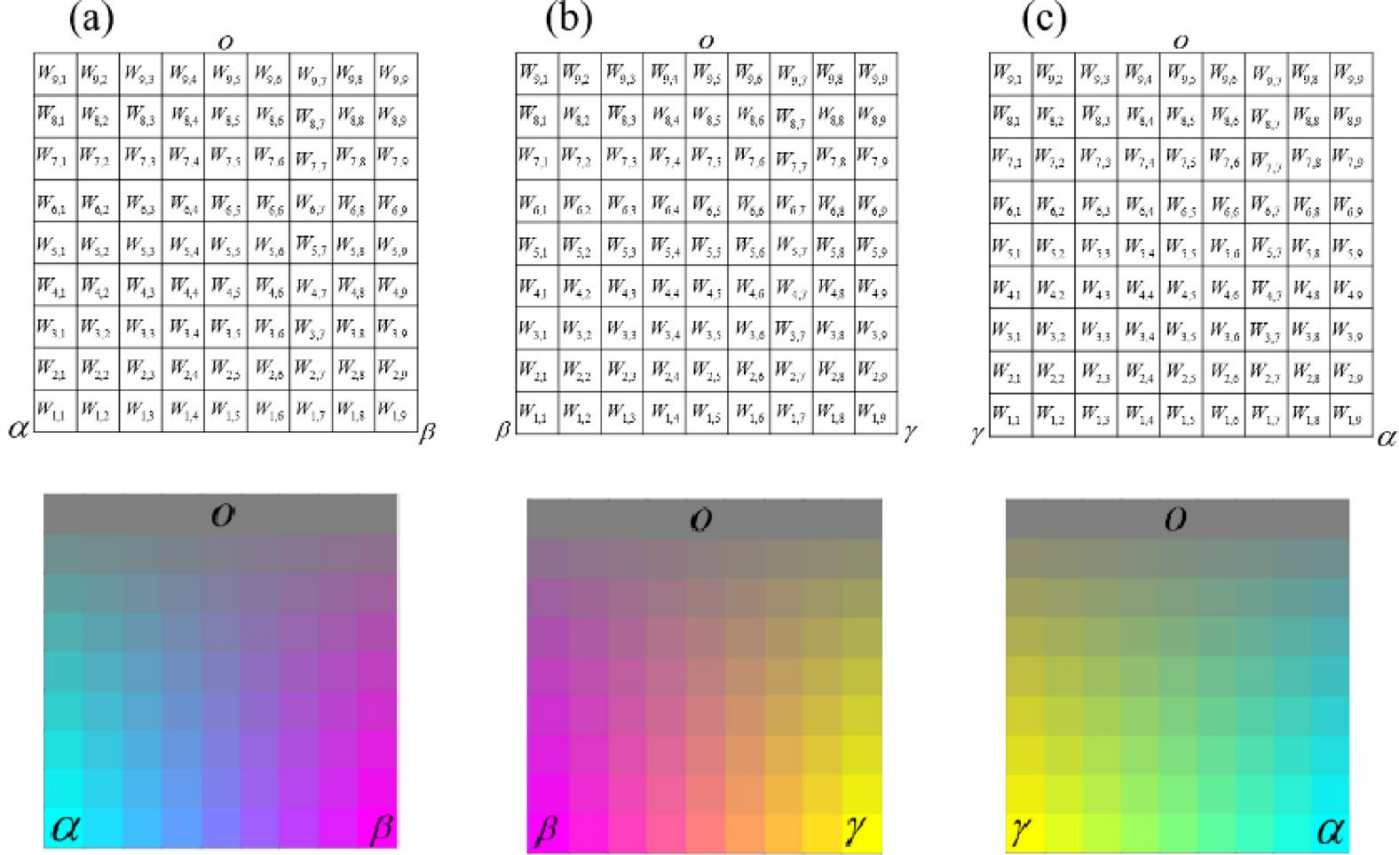
Three-panel visualization of the ternary dual-coupling color mixing models: (**a**) Cyan-Magenta-Grey (αβο), (**b**) Magenta-Yellow-Grey (βγο), and (**c**) Yellow-Cyan-Grey (γαο) grid structures.

The constructed ternary dual-coupling color mixing models are visualized in Fig. 1, which represents the fundamental submodels of our color mixing approach. As shown in Fig. 1, panel (a) depicts the αβο (Cyan-Magenta-Grey) three-color coupling, panel (b) shows the βγο (Magenta-Yellow-Grey) three-color coupling, and panel (c) illustrates the γαο (Yellow-Cyan-Grey) three-color coupling. Each grid structure consists of 9 × 9 = 81 network points, where the horizontal axis (j_2_) represents hue variation and the vertical axis (j_1_) represents saturation variation. The proportion of grey fiber increases from bottom to top, while the relative proportions of the two chromatic fibers change horizontally. This grid-based visualization demonstrates how systematic manipulation of mixing ratios produces predictable color variations across the spectrum, with each of the 81 points in each submodel representing a unique blend recipe. These three triangular planes serve as the building blocks for our comprehensive mixing model, providing localized color control within their respective domains before being integrated into the wide-color gamut system.

**Fig. 2:**
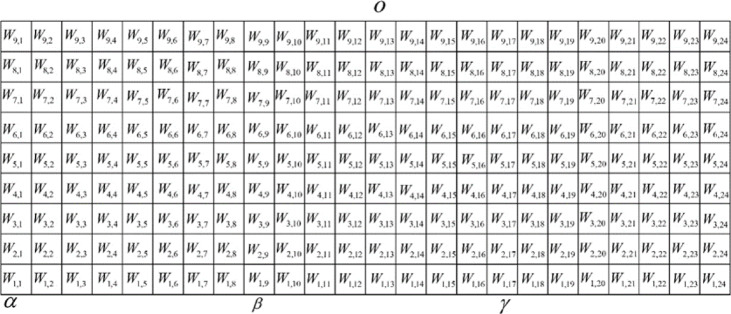
Integrated 216-point wide-color gamut mixing model created by interconnecting the three ternary dual-coupling systems across the α-β-γ-o color space.

The previously constructed coupling color mixing grid model contains 81 grid points. By varying the grid point coordinates, we can alter the mixing ratios of the four primary fibers (α, β, γ, o) and control hue, lightness, and saturation changes in three color domains: α-β-o, β-γ-o, and γ-α-o. However, these control methods are localized and cannot regulate color changes across the wide color gamut. To enable color regulation across the entire gamut and achieve precise digital control of hue, lightness, and saturation, it was necessary to construct a wide-color gamut grid-based mixing model. To accomplish this, we interconnected the three grid-based submodels from Fig. 1 by joining corresponding rows at their endpoints, creating a wide-color gamut grid-based mixing model incorporating the four primary colors: chromatic hues α, β, γ, and achromatic grey o. This comprehensive model, shown in Fig. 2, contains 216 grid points. By adjusting the grid point coordinates, we can modify the mixing ratios of the four primary fibers and uniformly control color changes in hue, lightness, and saturation across the entire α-β-γ-o color space.

### Three-channel numerical control spinning principles

**Fig. 3:**
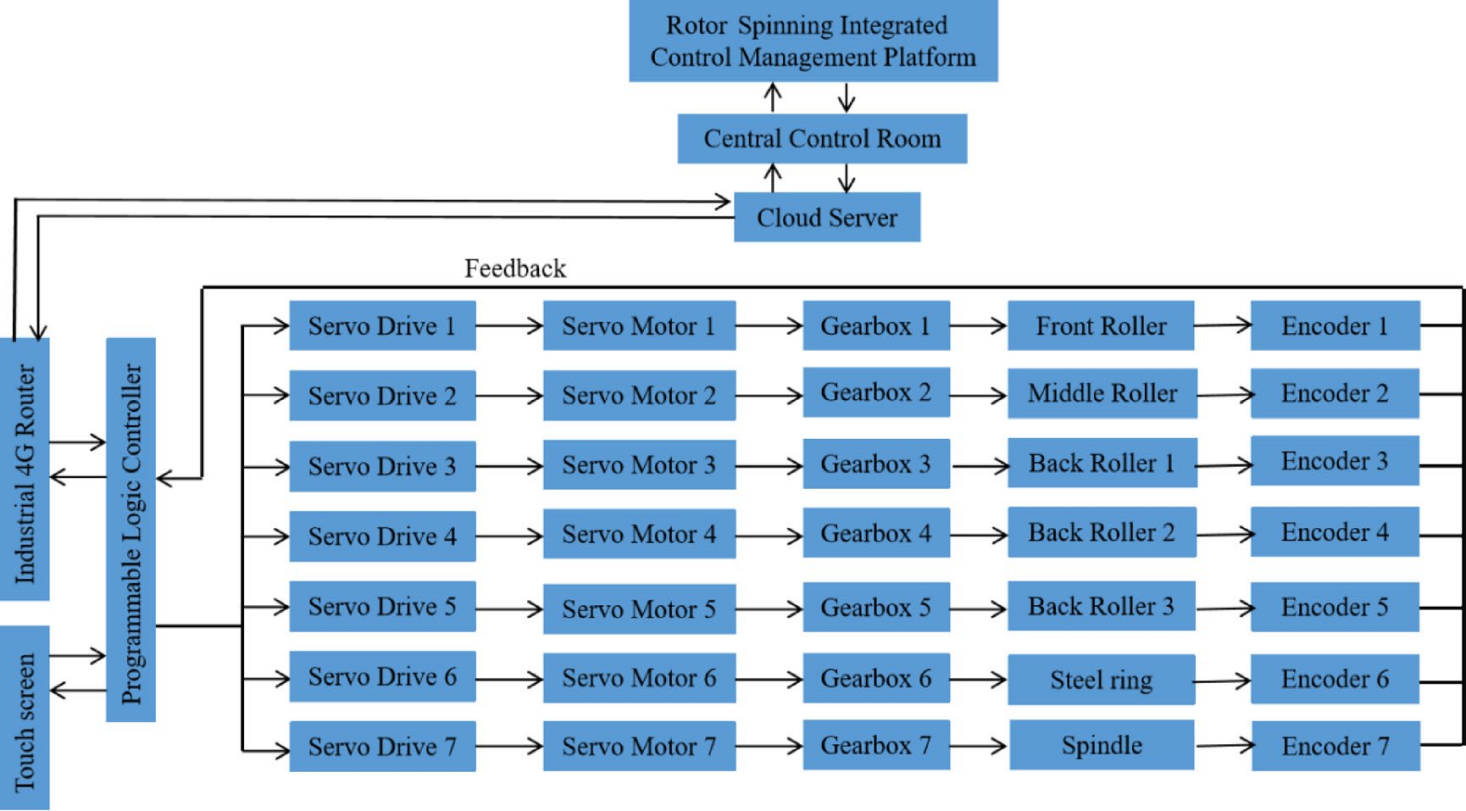
Programmable Logic Controller (PLC)-based control architecture of the three-channel numerical control rotor spinning system showing servo motors, interface and feedback mechanisms.

Three-channel numerical control spinning employs Programmable Logic Controller (PLC) technology to achieve real-time adjustment of the rotation speeds of servo motor-driven draft rollers in each channel, as shown in Fig. 3. This enables online control of three-channel roving draft ratios, the mixing ratios of three primary colored fibers in the forming yarn, and consequently the color of the forming yarn. By adjusting the three-channel roving draft ratios, we control the mixing ratios of three primary colored fibers, which in turn determines the color of the forming yarn. Conversely, we can also first design the yarn color values, then determine the required mixing ratios of three primary colored fibers, and subsequently determine the appropriate three-channel roving draft ratios.

**Fig. 4:**
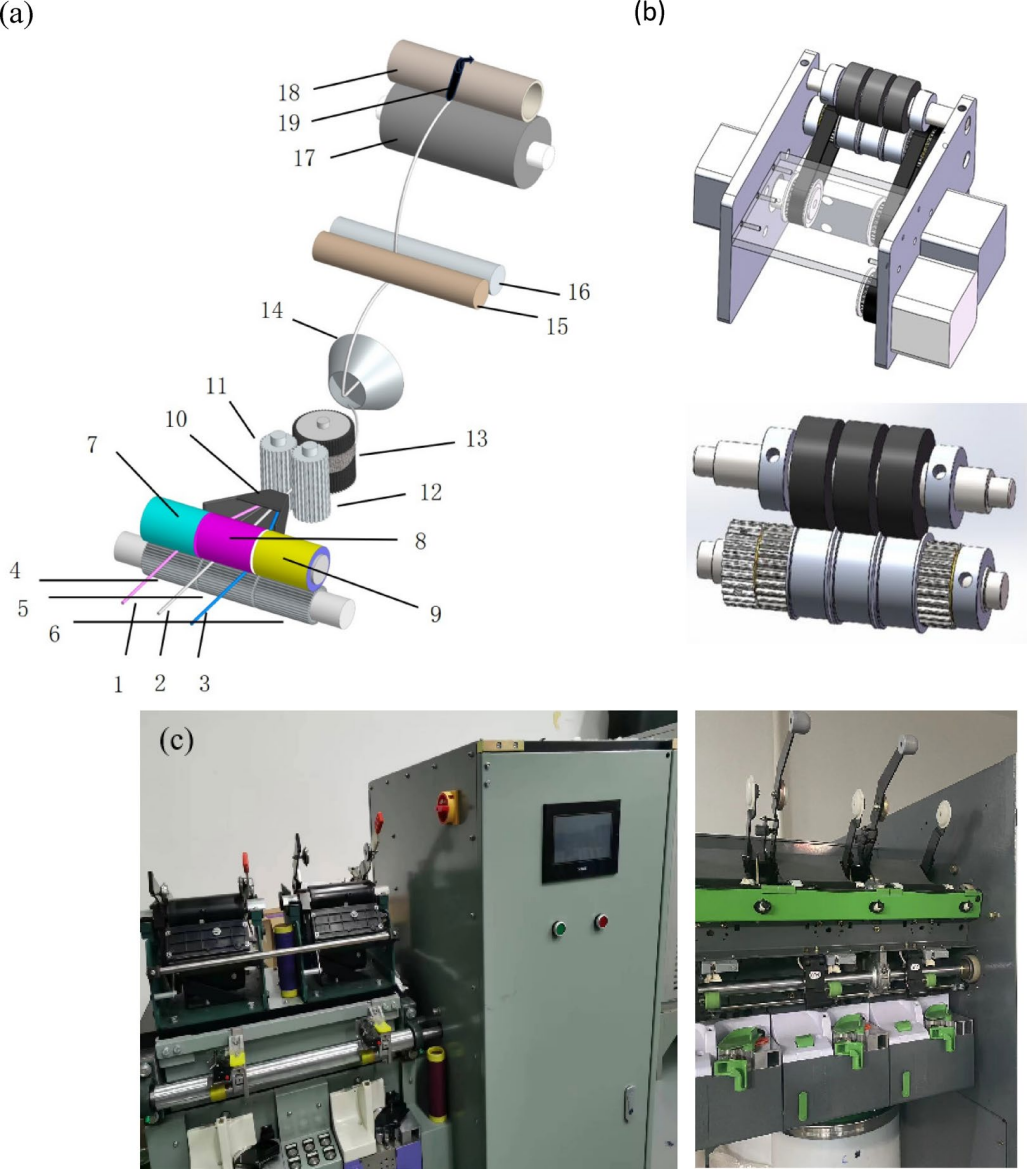
The three-channel computer numerical control (CNC) rotor spinning system. (**a**) Detailed schematic of the mechanical components and yarn path. The labels denote: (1, 2, 3) Input rovings; (4, 5, 6) Back rollers; (7, 8, 9) Back cots; (10) Fiber condenser; (11, 12) Middle rollers; (13) Combing roller; (14) Rotor; (15, 16) Take-up rollers; (17) Grooved drum; (18) Yarn package; (19) Traversing guide. (**b**) 3D mechanical drawing of the novel three-channel asynchronous feeding unit. (**c**) Photograph of the laboratory prototype machine used for the study.

The four primary fibers (α, β, γ, o) undergo opening, cleaning, carding, drawing, and roving processes to produce four primary-colored rovings with linear densities of ρ_α_, ρ_β_, ρ_γ_, and ρ_o_. The color values of these four rovings are C_α_(R_α_,G_α_,B_α_), C_β_(R_β_,G_β_,B_β_), C_γ_(R_γ_,G_γ_,B_γ_), and C_o_(R_o_,G_o_,B_o_). Using the three-channel digital spinning system shown in Fig. 4, we select any two from the three colored rovings α, β, γ, combined with the grey roving o, to form three base color rovings x, y, z. These are fed into independent drafting zones through their respective channel trumpet mouths, undergo front and back zone drafting, and then converge at the front nip point for twisting into yarn.

In the wide-color gamut model, where j_1_ = 1, 2, 3, …, 9 and δ = 1, 2, 3…, 23, 24, let the front roller linear speed be V_q_, the three back roller linear speeds be V_hx_(j_1_,δ), V_hy_(j_1_,δ), V_hz_(j_1_,δ), the three channel draft ratios be E_x_(j_1_,δ), E_y_(j_1_,δ), E_z_(j_1_,δ), the linear densities of three drafted slivers be ρ′_x_(j_1_,δ), ρ′_y_(j_1_,δ), ρ′_z_(j_1_,δ), the mixing ratios of each sliver in the yarn be λ_x_(j_1_,δ), λ_y_(j_1_,δ), λ_z_(j_1_,δ), the yarn linear density be ρ_s_, the yarn twist be T_w_, and the spindle speed be n_d_. In the three-channel color mixing numerical control spinning system shown in Fig. 4a, three rovings are fed through three independently driven back rollers and converge at the front roller nip point to enter the twisting mechanism. The linear density ρ_s_(j_1_,δ) of the resulting three-channel mixed color yarn is:

10 $${\uprho }_{\text{s}}({\text{j}}_{1},\updelta )=\left[\begin{array}{ccc}{\uprho }_{\text{x}}& {\uprho }_{\text{y}}& {\uprho }_{\text{z}}\end{array}\right]\left[\begin{array}{c}\frac{1}{{\text{E}}_{\text{x}}({\text{j}}_{1},\updelta )}\\ \frac{1}{{\text{E}}_{\text{y}}({\text{j}}_{1},\updelta )}\\ \frac{1}{{\text{E}}_{\text{z}}({\text{j}}_{1},\updelta )}\end{array}\right]={\uprho }_{\text{s}}({\text{j}}_{1},\updelta )\times \left[\begin{array}{c}{\uplambda }_{\text{x}}({\text{j}}_{1},\updelta )\\ {\uplambda }_{\text{y}}({\text{j}}_{1},\updelta )\\ {\uplambda }_{\text{z}}({\text{j}}_{1},\updelta )\end{array}\right]$$

where j_1_ = 1, 2, 3, …, 9 and δ = 1, 2, 3…, 23, 24.

Three asynchronously drafted slivers are combined and twisted to form yarn. The yarn mixing ratio is:

11 $$\left[\begin{array}{c}{\uplambda }_{\text{x}}({\text{j}}_{1},\updelta )\\ {\uplambda }_{\text{y}}({\text{j}}_{1},\updelta )\\ {\uplambda }_{\text{z}}({\text{j}}_{1},\updelta )\end{array}\right]=\left[\begin{array}{c}{\uprho }_{\text{x}}/{\text{E}}_{\text{x}}({\text{j}}_{1},\updelta )\times {\uprho }_{\text{s}}({\text{j}}_{1},\updelta )\\ {\uprho }_{\text{y}}/{\text{E}}_{\text{y}}({\text{j}}_{1},\updelta )\times {\uprho }_{\text{s}}({\text{j}}_{1},\updelta )\\ {\uprho }_{\text{z}}/{\text{E}}_{\text{z}}({\text{j}}_{1},\updelta )\times {\uprho }_{\text{s}}({\text{j}}_{1},\updelta )\end{array}\right]$$

where j_1_ = 1, 2, 3, …, 9 and δ = 1, 2, 3…, 23, 24.

Assuming the yarn color is determined by the color values of each roving and their mixing ratios, the yarn color C(j_1_,δ) = [C_r_, C_g_, C_b_]ᵀ is:

12 $$\text{C}({\text{j}}_{1},\updelta )=\left[\begin{array}{c}{\text{C}}_{\text{r}}({\text{j}}_{1},\updelta )\\ {\text{C}}_{\text{g}}({\text{j}}_{1},\updelta )\\ {\text{C}}_{\text{b}}({\text{j}}_{1},\updelta )\end{array}\right]=\left[\begin{array}{ccc}{\text{R}}_{\text{x}}& {\text{R}}_{\text{y}}& {\text{R}}_{\text{o}}\\ {\text{G}}_{\text{x}}& {\text{G}}_{\text{y}}& {\text{G}}_{\text{o}}\\ {\text{B}}_{\text{x}}& {\text{B}}_{\text{y}}& {\text{B}}_{\text{o}}\end{array}\right]\left[\begin{array}{c}{\uplambda }_{\text{x}}({\text{j}}_{1},\updelta )\\ {\uplambda }_{\text{y}}({\text{j}}_{1},\updelta )\\ {\uplambda }_{\text{o}}({\text{j}}_{1},\updelta )\end{array}\right]$$

where j_1_ = 1, 2, 3, …, 9 and δ = 1, 2, 3…, 23, 24.

The three-channel draft ratios are:

13 $$\left[\begin{array}{c}{\text{E}}_{\text{x}}({\text{j}}_{1},\updelta )\\ {\text{E}}_{\text{y}}({\text{j}}_{1},\updelta )\\ {\text{E}}_{\text{z}}({\text{j}}_{1},\updelta )\end{array}\right]={\left[\begin{array}{ccc}{\uplambda }_{\text{x}}({\text{j}}_{1},\updelta )& 0& 0\\ 0& {\uplambda }_{\text{y}}({\text{j}}_{1},\updelta )& 0\\ 0& 0& {\uplambda }_{\text{z}}({\text{j}}_{1},\updelta )\end{array}\right]}^{-1}\left[\begin{array}{c}{\uplambda }_{\text{x}}({\text{j}}_{1},\updelta )\\ {\uplambda }_{\text{y}}({\text{j}}_{1},\updelta )\\ {\uplambda }_{\text{z}}({\text{j}}_{1},\updelta )\end{array}\right]$$

where j_1_ = 1, 2, 3, …, 9 and δ = 1, 2, 3…, 23, 24.

Assuming the yarn color is C(j_1_,δ) = [C_r_, C_g_, C_b_]ᵀ, and the colors of the three-channel rovings are C_x_(R_x_, G_x_, B_x_), C_y_(R_y_, G_y_, B_y_), and C_z_(R_z_, G_z_, B_z_), the mixing ratio parameters from Eq. (13) are:

14 $$\left[\begin{array}{c}{\uplambda }_{\text{x}}({\text{j}}_{1},\updelta )\\ {\uplambda }_{\text{y}}({\text{j}}_{1},\updelta )\\ {\uplambda }_{\text{z}}({\text{j}}_{1},\updelta )\end{array}\right]={\left[\begin{array}{ccc}{\text{R}}_{\text{x}}& {\text{R}}_{\text{y}}& {\text{R}}_{\text{o}}\\ {\text{G}}_{\text{x}}& {\text{G}}_{\text{y}}& {\text{G}}_{\text{o}}\\ {\text{B}}_{\text{x}}& {\text{B}}_{\text{y}}& {\text{B}}_{\text{o}}\end{array}\right]}^{-1}\left[\begin{array}{c}{\text{C}}_{\text{R}}({\text{j}}_{1},\updelta )\\ {\text{C}}_{\text{G}}({\text{j}}_{1},\updelta )\\ {\text{C}}_{\text{B}}({\text{j}}_{1},\updelta )\end{array}\right]$$

where j_1_ = 1, 2, 3, …, 9 and δ = 1, 2, 3…, 23, 24.

From Eq. (11), and assuming ρ_x_ = ρ_y_ = ρ_z_ = ρ, the three-channel draft ratios can be determined as:

15 $$\text{E}({\text{j}}_{1},\updelta )=\left[\begin{array}{c}{\text{E}}_{\text{x}}({\text{j}}_{1},\updelta )\\ {\text{E}}_{\text{y}}({\text{j}}_{1},\updelta )\\ {\text{E}}_{\text{z}}({\text{j}}_{1},\updelta )\end{array}\right]=\frac{\uprho }{{\uprho }_{\text{s}}}\times \left[\begin{array}{c}\frac{1}{{\uplambda }_{\text{x}}({\text{j}}_{1},\updelta )}\\ \frac{1}{{\uplambda }_{\text{y}}({\text{j}}_{1},\updelta )}\\ \frac{1}{{\uplambda }_{\text{z}}({\text{j}}_{1},\updelta )}\end{array}\right]$$

where j_1_ = 1, 2, 3, …, 9 and δ = 1, 2, 3…, 23, 24.

### Three-channel CNC rotor spinning system: design and operating principle

The experimental yarns were produced on a custom-built three-channel CNC rotor spinning system. This system was developed by retrofitting a commercial TQF-K80 rotor spinning machine (Zhejiang Taitan Co., Ltd.) with a novel three-channel asynchronous feeding apparatus and an expanded nine-axis servo-control system. The target yarn count for all 90 experimental samples was 32 tex with a twist of 800 twists/meter, spun from three input rovings.

The mechanical layout of the custom-built three-channel rotor spinning system is detailed in Fig. 4. As shown in the schematic in Fig. 4a, the process begins with three separate rovings (1, 2, 3) being fed into the system by three independent back rollers (4, 5, 6) and their corresponding back cots (7, 8, 9). These rovings are initially drafted between the back rollers and a common pair of middle rollers (11, 12). The three drafted fiber streams then pass through a fiber condenser (10) before converging and being fed into a single high-speed combing roller (13). This roller performs the critical dual function of opening the rovings into individual fibers while simultaneously ensuring a thorough and uniform mixing of the fibers from the three different channels. This homogeneous fiber blend is then transported via airflow into the rotor (14), where it is collected in the rotor groove and twisted into the final yarn. The finished yarn is withdrawn by take-up rollers (15, 16) and wound by a grooved drum (17) and traversing guide (19) onto a yarn package (18). The core innovation of this system is the digitally controlled, three-channel feeding unit, which is shown in the 3D mechanical drawing in Fig. 4b and as a physical prototype in Fig. 4c. It consists of three nested, coaxial back rollers, each driven by an independent servo motor. This design is the key mechanism that allows precise, real-time control over the feed rate of each individual roving, thereby enabling dynamic adjustment of the fiber blend ratio and, consequently, the final yarn color. Each roving is guided through independent trumpet guides into its respective drafting zone, maintaining consistent geometric relationships and preventing interference during feeding.

The effectiveness of this system for producing uniformly blended color-spun yarns is rooted in the fundamental fiber integration mechanism of rotor spinning. Unlike ring spinning, where the geometry of the spinning triangle can lead to incomplete mixing, the rotor spinning process inherently randomizes fiber distribution. After being opened by the combing roller, the three fiber streams are transported by airflow and deposited by centrifugal force into the rotor groove. Here, they are systematically re-integrated and layered onto the rotating yarn tail. A critical consideration in any multi-component spinning system is whether the initial feeding position of the rovings influences the final yarn properties. In our system, this effect is negligible for the final perceived color due to the superior blending efficiency of the rotor. The randomization process ensures a highly homogeneous radial and longitudinal distribution of the different colored fibers, as confirmed by microscopic analysis (Fig. 8). Therefore, the excellent agreement between our predicted and experimental color values is predicated on this blending efficiency, which makes the final color dependent only on the digitally controlled fiber proportions, not their initial channel.

To ensure consistency and reproducibility across all 90 experimental samples, rigorous standardization protocols were implemented. All primary-colored rovings were produced with a consistent twist of 68 twists/10 cm, and the total feed rate of the three channels was maintained at 2 m/min. This ensured that all yarns were produced under identical and stable conditions, providing a reliable basis for validating our color prediction model.

### Process design for spinning color yarn based on wide-color gamut mixing model

**Fig. 5:**
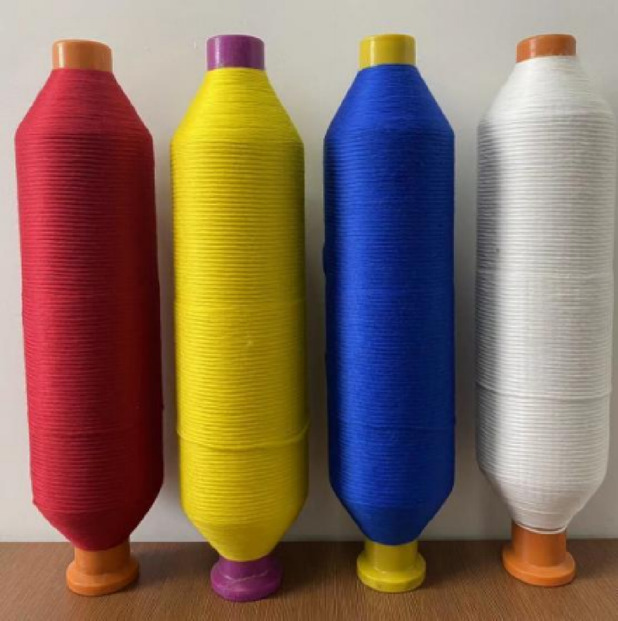
Schematic of the four primary-colored rovings with a standardized linear density of 450 tex (0.45 g/m) and a twist of 680 tpm.

To implement and validate the theoretical color mixing model developed in “Design and construction of the wide-gamut color mixing system”, we used rovings made from 100% Xinjiang fine-staple cotton (Grade 2, saw-ginned, with a staple length of 29 mm and a Micronaire value of Grade A), sourced from Anhui Chaohu Youngor Color Spinning Co., Ltd. The raw cotton was dyed in four primary colors—Red, Yellow, Blue, and Grey—and processed through opening, carding, drawing, and roving to produce rovings with a standardized linear density of 450 tex and a twist of 680 twists per meter (tpm). The key advantage of our model is its generalizability, as its inputs are the final, measured RGB color values of the rovings (Table 1), not the specific dyeing parameters used to achieve them. This design makes our digital color-mixing system independent of the upstream dyeing process (e.g., dye class or shade depth), ensuring its robust applicability across different raw materials and industrial conditions, as long as the primary colors can be instrumentally quantified. Using a 3nh YS6010 spectrophotometer under D65 illuminant, 10° observer field, 25.4 mm aperture, and Specular Component Included (SCI) mode, we measured the color values of the four primary colors. Each sample was measured five times to determine the average values, as shown in Table 1. Figure 5 shows a schematic diagram of the four primary color rovings.

**Table 1:** Measured RGB color values of the four experimental primary rovings.

Roving color	Red	Yellow	Blue	Grey
RGB color	RGB:145 37 49	RGB:246 199 0	RGB:3 78 135	RGB:240 237 232
Simulated color

**Fig. 6:**
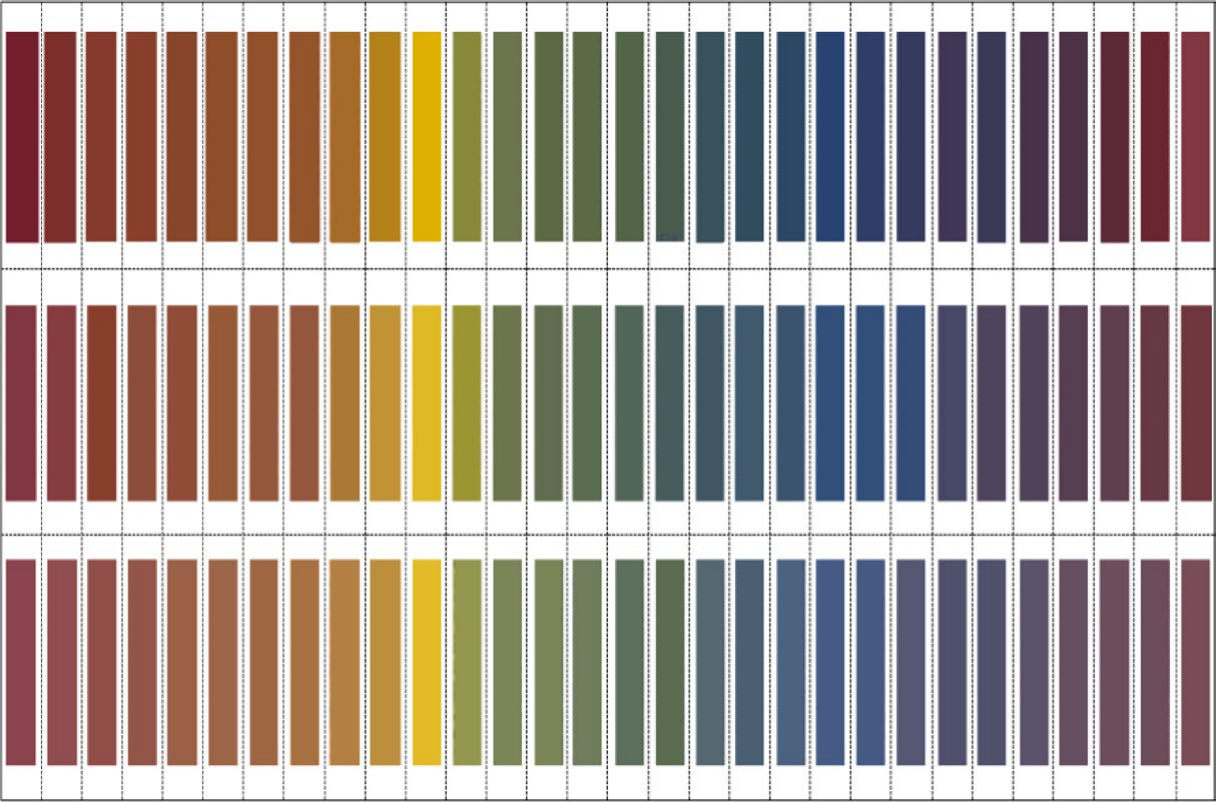
Visualization of wide-gamut panchromatic color models derived from four primary colors (red, yellow, blue, grey) showing both circular spectrum representation and rectangular color space mapping of achievable yarn colorations.

Using the four primary color mixing scheme, we developed a panchromatic color ring spectrum and rectangular color spectrum based on red (RGB:145 37 49), yellow (RGB:246 199 0), blue (RGB:3 78 135), and grey (RGB:240 237 232) colors, as shown in Fig. 6. To clearly represent the 90 experimental objectives, we selected 90 experimental grid points from the panchromatic mixing ratio table, which are marked in red in Table 2. For our experiments, we established mechanical parameters to facilitate the calculation of process parameters for spinning experiments. We selected a roving linear density of 450 tex, set the sum of the three feeding roller speeds at 2 m/min, yarn drawing speed at 18 m/min, carding roller speed at 6000 r/min, rotor speed at 20,000 r/min, and suction frequency at 45 Hz, as shown in Table 3. These speeds fall within the recommended operating ranges for the HFX-03-T prototype machine used in this study. The relatively high carding roller speed was determined to be optimal for ensuring both sufficient fiber opening and the homogeneous blending of the three separate input rovings, a critical requirement for uniform color distribution.

**Table 2:**
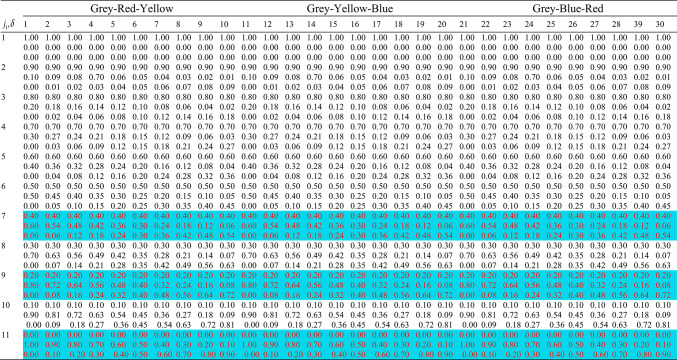
Wide-color gamut of 90 experimental subjects.

**Table 3:** Rotor yarn process design parameters.

Linear density of roving	Sum of three feeding speeds	Yarn drawing speed	Carding roller speed	Rotor speed
450 g/100 m	2 m/min	18 m/min	6000 r/min	20,000 r/min

In this study, 90 different yarn samples were produced using the three-channel rotor spinning system. These 90 yarns were created by combining red, yellow, blue, and grey primary colors in various proportions, using gradients of 0.1, 0.08, and 0.06. To ensure the reliability and comparability of all physical property data, the yarn samples were conditioned prior to testing. All yarns were placed in a standard atmosphere of 20 ± 2 °C and 65 ± 5% relative humidity for 48 h, in accordance with ISO 139:2005 “Textiles—Standard atmospheres for conditioning and testing”. We used a YG133B/PRO-H evenness tester (Suzhou Changfeng Textile Electromechanical Technology Co., Ltd.) in accordance with ISO 16,549–2021 “Unevenness of textile strands—Capacitance method” at a speed of 200 m/min with a 2.5 min testing time. We used a YG172A yarn hairiness tester (Shaanxi Changling Textile Electromechanical Technology Co., Ltd.) in accordance with FZ/T 01,086–2000 " Test Method for Yarn Hairness—Projection Counting Method" at a speed of 30 m/s. The working principle of this device is the projection counting method, which is crucial for ensuring measurement consistency across different colored yarns. In this system, the yarn passes through a field of uniform, monochromatic light, casting a shadow onto a linear photoelectric sensor. The instrument’s electronics differentiate the continuous shadow of the yarn’s core from the intermittent shadows of the protruding hairs. Because the measurement is based on the occlusion of a single wavelength of light, the different colors of the yarns do not influence the results; all yarns are treated as opaque bodies. This principle ensures that the hairiness data is comparable and reliable across all 90 experimental samples. The test length for each replication was set to 10 m, as stipulated by the FZ/T 01086-2000 standard for cotton-type yarns, to ensure a consistent and reproducible baseline for the comparative analysis across all 90 samples. Three tests were performed for each yarn type to obtain an average value. According to the standard, hairs longer than 3 mm are considered as the setting length for cotton yarns. We used an XL-2 yarn strength tester (Shanghai Xinxian Instruments Co., Ltd.) in accordance with ISO 2062–2009 "Textiles—Yarns from packages—Determination of single-end breaking force and elongation at break using constant rate of extension (CRE) tester" at a test speed of 500 mm/min, performing 10 tests to obtain an average value. Testing parameters included a pre-tension of 16 cN and clamping distance of 500 mm.

After producing the yarns, we selected the blue-grey-red series as test subjects for comprehensive performance evaluation. The yarns were also knitted into fabric swatches using a 16G Hongcheng HC21K type weft circular knitting machine with the following specifications: loop length of 0.6 mm, wale density of 10.6 wales/cm, and course density of 18 courses/cm. The fabrics were scanned and their color values were measured using a 3nh YS6010 spectrophotometer under D65 illuminant, 10° observer field, 25.4 mm aperture, and SCI mode.

## Results and discussion

**Fig. 7:**
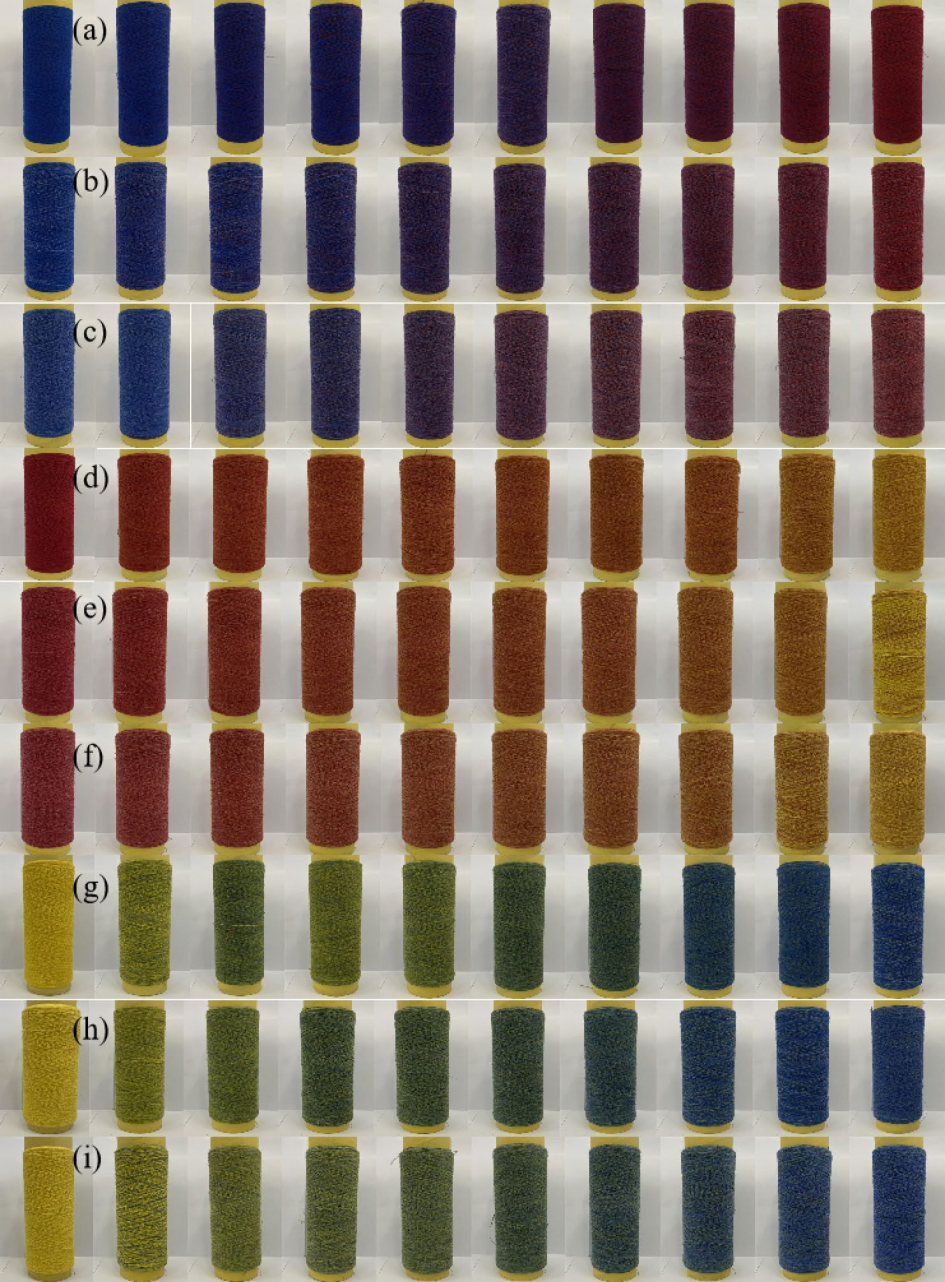
(**a**–**i**) Photographic overview of the 90 digitally spun yarns, organized into three test series.

As shown in Fig. 7, we successfully produced 90 different yarn samples using the three-channel rotor spinning system. These 90 yarns represent 90 different mixing ratios, 90 different feeding speeds, and 90 different color values, arranged according to the color and mixing ratio of the input rovings. The yarns display rich and vibrant colors, achieving the requirement of producing colorful and harmonious blended yarns through rotor spinning. We conducted extensive spinning experiments with different fiber combinations: Blue-grey-red series with 0.1, 0.08, and 0.06 gradients (Fig. 7a–c); Yellow-grey-red series with 0.1, 0.08, and 0.06 gradients (Fig. 7d–f)); Yellow-grey-blue series with 0.1, 0.08, and 0.06 gradients (Fig. 7g–i). The experimental results showed successful production of all designed color variations, with visible gradient changes across each series. More details of experimental scheme are found in supplemental Table S1–S9.

**Fig. 8:**
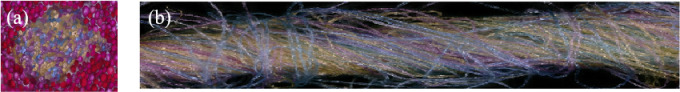
Microscopic analysis of digitally controlled color-spun yarn structure: (**a**) Cross-sectional view showing uniform radial distribution of four primary-colored fibers at 50 × magnification; (**b**) Longitudinal view demonstrating helical fiber arrangement characteristic of rotor-spun structure.

To validate the physical realization of our theoretical color mixing model, we conducted comprehensive microscopic examination of representative yarn samples using a VHX-5000 ultra-depth 3D microscope (Keyence Corporation) at 50 × magnification. Figure 8 presents both cross-sectional and longitudinal views of digitally controlled color-spun yarns produced using our three-channel system. The cross-sectional images (Fig. 8a) reveal uniform radial distribution of the four primary-colored fibers (cyan, magenta, yellow, and grey) throughout the yarn cross-section, confirming the effectiveness of our digital control system in achieving homogeneous fiber blending. No evidence of fiber segregation or clustering was observed, which could otherwise compromise color uniformity and prediction accuracy. The longitudinal view (Fig. 8b) demonstrates the characteristic helical arrangement of fibers around the yarn axis, typical of rotor-spun structure, with all four fiber types contributing to both structural integrity and color formation. This microscopic evidence provides crucial validation that our mathematical color prediction model accurately represents the physical fiber mixing process. The uniform fiber distribution observed supports our fundamental assumption that yarn color values can be calculated using weighted averages of primary fiber RGB values according to their respective blending ratios (Eq. 9). The consistent fiber arrangement throughout the yarn length confirms that our three-channel digital control system maintains stable blending ratios during continuous spinning, thereby ensuring reproducible color outcomes.

The mechanical properties and surface characteristics of the digitally controlled color-spun yarns were comprehensively analyzed to assess the relationship between fiber blend ratios and resultant yarn performance. It is well-established that the blend ratio of constituent fibers is a dominant factor influencing the final mechanical and structural properties of a yarn, as it governs both the overall blending uniformity and the microscopic arrangement of fibers within the yarn structure, such as fiber migration and packing density^25,36^. Analysis of variance (ANOVA) confirmed that the blend composition had a statistically significant effect on all measured mechanical and evenness metrics (*p* < 0.001 for all variables), validating that the observed variations are due to the blend ratios and not random chance. Table 4 presents the mechanical properties and evenness data, revealing significant correlations between blend compositions and yarn quality metrics. Tenacity values exhibit considerable variation (7.10–13.40 cN/tex), demonstrating the profound impact of fiber composition on mechanical performance. Notably, balanced compositions perform exceptionally well, with the 0.40:0.20:0.40 (blue:grey:red) blend achieving the highest tenacity (13.40 cN/tex), indicating optimal fiber interaction when chromatic components are balanced with moderate grey content. Post-hoc analysis using Tukey’s HSD test revealed that blends containing moderate grey proportions (0.20–0.40) consistently produced significantly higher tenacity values (*p* < 0.05) compared to yarns with no grey content. When combined with balanced chromatic components, these moderate grey proportion blends achieved superior tenacity values ranging from 10.0 to 13.40 cN/tex, consistently exceeding the industry standard threshold of 9.0 cN/tex for Grade 2 quality according to FZ/T 12001-2015.

**Table 4:** Mechanical properties and evenness metrics for the panchromatic blended yarns. The coefficient of variation (CV%) for each measurement is shown in parentheses.

Blend ratio (blue : grey : red)	Breaking force (cN)	Tenacity (cN/tex)	Initial modulus (cN/tex)	Elongation at break (%)	Evenness U (%)	Coefficient of variation of yarn Mass (CVm %)
0.60:0.40:0.00	380.9 (4.9%)	11.40 (5.1%)	141.90 (6.8%)	0.40 (8.5%)	19.15 (2.5%)	24.24 (2.8%)
0.54:0.40:0.06	314.2 (6.1%)	9.20 (6.3%)	168.60 (7.5%)	0.30 (10.2%)	16.45 (2.1%)	22.46 (2.4%)
0.48:0.40:0.12	351.3 (5.5%)	10.30 (5.7%)	153.30 (7.1%)	0.40 (8.9%)	26.17 (3.1%)	32.69 (3.5%)
0.42:0.40:0.18	396.4 (4.5%)	11.70 (4.7%)	124.90 (6.2%)	0.40 (8.2%)	17.92 (2.3%)	22.79 (2.6%)
0.36:0.40:0.24	353.3 (5.2%)	10.40 (5.4%)	116.50 (6.5%)	0.40 (8.6%)	13.07 (1.8%)	16.81 (2.0%)
0.30:0.40:0.30	356.6 (5.0%)	10.50 (5.2%)	136.10 (6.9%)	0.40 (8.4%)	11.20 (1.5%)	14.52 (1.7%)
0.24:0.40:0.36	431.7 (4.1%)	12.70 (4.3%)	80.80 (8.8%)	0.50 (7.1%)	15.84 (2.0%)	20.43 (2.3%)
0.18:0.40:0.42	380.9 (4.8%)	11.40 (5.0%)	128.70 (6.6%)	0.40 (8.3%)	16.40 (2.1%)	21.22 (2.4%)
0.12:0.40:0.48	297.8 (6.5%)	8.80 (6.8%)	160.20 (7.8%)	0.30 (10.8%)	16.40 (2.1%)	21.22 (2.4%)
0.12:0.40:0.48	297.8 (6.5%)	8.80 (6.8%)	160.20 (7.8%)	0.30 (10.8%)	16.40 (2.1%)	21.22 (2.4%)
0.06:0.40:0.54	318.8 (5.9%)	9.40 (6.1%)	136.70 (7.3%)	0.30 (10.5%)	19.76 (2.6%)	27.02 (3.0%)
0.80:0.20:0.00	304.3 (6.2%)	8.90 (6.4%)	121.80 (8.0%)	0.30 (10.6%)	11.73 (1.6%)	15.21 (1.8%)
0.72:0.20:0.08	268.7 (7.1%)	7.90 (7.4%)	147.80 (8.5%)	0.20 (12.5%)	13.97 (1.9%)	17.75 (2.1%)
0.64:0.20:0.16	341.8 (5.4%)	10.10 (5.6%)	99.30 (8.2%)	0.40 (8.7%)	12.84 (1.7%)	16.54 (1.9%)
0.56:0.20:0.24	393.1 (4.6%)	11.60 (4.8%)	121.70 (6.4%)	0.40 (8.1%)	12.11 (1.6%)	15.43 (1.8%)
0.48:0.20:0.32	341.1 (5.5%)	10.00 (5.8%)	123.20 (7.9%)	0.40 (8.8%)	17.78 (2.3%)	24.38 (2.7%)
0.40:0.20:0.40	454.1 (3.9%)	13.40 (4.0%)	114.10 (6.0%)	0.60 (6.5%)	14.12 (1.9%)	18.19 (2.1%)
0.32:0.20:0.48	357.1 (5.1%)	10.50 (5.3%)	130.90 (7.0%)	0.40 (8.5%)	10.27 (1.4%)	13.20 (1.6%)
0.24:0.20:0.56	292.9 (6.6%)	8.60 (6.9%)	160.80 (8.1%)	0.30 (11.0%)	13.18 (1.8%)	17.99 (2.0%)
0.16:0.20:0.64	309.1 (6.0%)	9.10 (6.2%)	154.60 (8.3%)	0.30 (10.7%)	14.94 (2.0%)	19.05 (2.2%)
0.08:0.20:0.72	268.8 (7.0%)	7.90 (7.3%)	167.80 (8.6%)	0.20 (12.8%)	14.57 (1.9%)	18.80 (2.2%)
1.00:0.00:0.00	333.0 (5.6%)	9.80 (5.8%)	142.90 (7.6%)	0.30 (10.1%)	19.30 (2.5%)	31.58 (3.6%)
0.90:0.00:0.10	241.1 (7.8%)	7.10 (8.1%)	177.00 (9.0%)	0.20 (13.5%)	17.52 (2.2%)	22.53 (2.6%)
0.80:0.00:0.20	262.9 (7.2%)	7.70 (7.5%)	180.50 (9.2%)	0.20 (13.1%)	15.75 (2.0%)	20.46 (2.3%)
0.70:0.00:0.30	323.3 (5.8%)	9.50 (6.0%)	134.60 (7.4%)	0.30 (10.3%)	20.32 (2.6%)	25.26 (2.9%)
0.60:0.00:0.40	396.0 (4.5%)	11.60 (4.7%)	107.60 (6.8%)	0.40 (8.0%)	10.95 (1.5%)	14.22 (1.7%)
0.50:0.00:0.50	402.6 (4.4%)	11.80 (4.6%)	114.60 (6.5%)	0.40 (7.9%)	15.17 (2.0%)	19.65 (2.3%)
0.40:0.00:0.60	252.1 (7.5%)	7.40 (7.8%)	173.40 (9.1%)	0.20 (13.3%)	17.17 (2.2%)	22.10 (2.5%)
0.30:0.00:0.70	280.4 (6.8%)	8.20 (7.1%)	170.40 (8.9%)	0.30 (11.5%)	30.27 (3.5%)	38.81 (4.1%)

When analyzing tenacity against blue fiber content while maintaining a constant grey proportion (0.40), regression analysis confirmed the observed parabolic relationship (R^2^ = 0.76, *p* < 0.01), with the model predicting optimal strength at mid-range blue concentrations rather than at extremes. This suggests that the grey fiber acts as a structural intermediary between the chromatic components, with optimal grey concentration creating ideal conditions for fiber cohesion and load transfer. The initial modulus data (80.80–180.50 cN/tex) reveals complementary insights into yarn stiffness, often showing an inverse relationship with tenacity. For example, the 0.80:0.00:0.20 blend exhibits high modulus (180.50 cN/tex) but relatively low tenacity (7.70 cN/tex), suggesting brittle behavior with limited capacity for energy absorption before failure. In contrast, best-performing yarns (tenacity > 11.0 cN/tex) typically demonstrate moderate modulus values (80.80–141.90 cN/tex), indicating an ideal balance between rigidity and deformation capacity.

The elongation at break data further clarifies the mechanical profile, showing a strong positive correlation with tenacity and an inverse relationship with initial modulus. For instance, the blend with the highest tenacity (0.40:0.20:0.40) also exhibited the highest elongation at break (0.60%), demonstrating superior ductility. In contrast, blends with a high initial modulus, such as the 0.80:0.00:0.20 composition, were correspondingly brittle, with a low elongation of just 0.20%. This trend confirms that the balanced blend compositions identified as optimal not only possess high strength but also greater toughness, representing a more desirable overall performance for most textile applications.

The evenness metrics demonstrate significant variation across blend compositions, with U% values ranging from 10.27% to 30.27% and corresponding CVm values from 13.20 to 38.81%. It is important to note that the higher CVm values are an expected outcome of testing the system’s limits with extreme, non-optimized blend ratios. This variation confirms that blend ratios profoundly affect structural uniformity. A critical threshold effect is observed when any component proportion exceeds approximately 0.70. This is statistically evident in the 0.30:0.00:0.70 blend, whose U% of 30.27% ± 1.85% was significantly higher than all other tested compositions (*p* < 0.001). The most uniform structures (U% < 12%) occur in blends with grey proportions between 0.20 and 0.40 and balanced chromatic components, paralleling the patterns observed for mechanical properties. These findings indicate that extreme blend ratios create processing challenges during drafting and fiber alignment, resulting in structural irregularities manifested as elevated U% and CVm values. Conversely, the most uniform structures occur in blends with more balanced component proportions. For instance, the 0.32:0.20:0.48 blend achieved a CVm of 13.20%, and the high-tenacity 0.40:0.20:0.40 blend had a CVm of 18.19%. These values are well within the Grade 2 quality threshold (CVm < 21%) specified by the FZ/T 12001-2015 standard for rotor-spun yarns of this count, confirming that the system can produce high-quality yarn when operating with optimized blend parameters.

Table 5 provides critical insights into surface characteristics through hairiness profiles across different blend ratios. ANOVA results indicated that while short hairiness (≥ 1 mm) did not vary significantly across most compositions (*p* > 0.05), longer hairiness (≥ 3 mm and ≥ 4 mm) was significantly influenced by the blend ratio (*p* < 0.01). This suggests that primary hairiness formation mechanisms affect all blends similarly, while secondary mechanisms governing longer, more problematic hairs are composition-dependent. For instance, the 0.30:0.40:0.30 blend exhibited a statistically higher count of medium-length hairiness (≥ 3 mm: 41.3 ± 3.5 hairs/10 m) compared to other blends in its series. For blends with constant grey content, a significant positive correlation was found between increasing red content and long hairiness (≥ 4 mm) (Pearson’s r = 0.62, *p* < 0.05), which suggests that the characteristics of the red fiber promote surface protrusion.

**Table 5:** Hairiness profile of color blend yarns (hairs ≥ n mm per 10 m), with measurement CV% in parentheses.

Blend ratio (Blue : grey : Red)	≥ 1 mm	≥ 2 mm	≥ 3 mm	≥ 4 mm	≥ 5 mm	≥ 6 mm
0.60:0.40:0.00	432.6 (6.8%)	110.6 (8.5%)	35.0 (10.8%)	10.00 (15.5%)	4.67 (21.3%)	1.67 (35.8%)
0.54:0.40:0.06	361.0 (7.5%)	75.0 (9.8%)	22.6 (13.1%)	9.67 (16.0%)	5.33 (20.1%)	1.00 (45.0%)
0.48:0.40:0.12	396.6 (7.1%)	90.00 (9.1%)	33.6 (11.2%)	10.67 (15.1%)	6.00 (19.0%)	1.33 (40.1%)
0.42:0.40:0.18	410.6 (6.9%)	103.3 (8.8%)	30.3 (11.8%)	11.00 (14.9%)	4.33 (22.5%)	3.00 (28.1%)
0.36:0.40:0.24	386.0 (7.3%)	86.33 (9.4%)	28.6 (12.3%)	10.67 (15.2%)	4.33 (22.5%)	2.00 (33.5%)
0.30:0.40:0.30	435.3 (6.7%)	107.3 (8.6%)	41.3 (10.1%)	17.67 (12.5%)	6.67 (18.1%)	2.67 (29.9%)
0.24:0.40:0.36	406.0 (7.0%)	98.00 (9.0%)	36.3 (10.6%)	14.67 (13.5%)	6.33 (18.6%)	2.67 (29.9%)
0.18:0.40:0.42	410.6 (6.9%)	94.67 (9.2%)	34.0 (11.0%)	9.67 (15.8%)	3.67 (24.5%)	1.33 (40.1%)
0.12:0.40:0.48	338.6 (7.8%)	83.33 (9.6%)	31.3 (11.6%)	9.33 (16.2%)	3.00 (27.7%)	1.67 (35.8%)
0.06:0.40:0.54	436.6 (6.7%)	96.00 (9.1%)	35.6 (10.7%)	11.67 (14.5%)	8.33 (16.8%)	3.00 (28.1%)
0.80:0.20:0.00	457.0 (6.5%)	105.3 (8.7%)	39.3 (10.3%)	12.67 (14.1%)	6.33 (18.6%)	1.00 (45.0%)
0.72:0.20:0.08	424.0 (6.9%)	92.33 (9.2%)	31.0 (11.7%)	11.00 (14.9%)	5.67 (19.5%)	2.33 (31.5%)
0.64:0.20:0.16	493.0 (6.1%)	113.3 (8.4%)	36.6 (10.5%)	9.33 (16.2%)	2.67 (29.9%)	2.00 (33.5%)
0.56:0.20:0.24	364.6 (7.5%)	81.33 (9.7%)	29.3 (12.1%)	9.67 (15.9%)	6.00 (19.0%)	1.67 (35.8%)
0.48:0.20:0.32	420.0 (6.9%)	110.3 (8.5%)	33.0 (11.3%)	10.33 (15.4%)	5.00 (20.8%)	1.67 (35.8%)
0.40:0.20:0.40	421.0 (6.8%)	103.3 (8.8%)	32.6 (11.4%)	10.67 (15.2%)	6.00 (19.0%)	1.67 (35.8%)
0.32:0.20:0.48	379.6 (7.3%)	79.00 (9.8%)	26.6 (12.6%)	11.33 (14.7%)	3.67 (24.5%)	1.33 (40.1%)
0.24:0.20:0.56	354.6 (7.6%)	85.33 (9.5%)	32.6 (11.4%)	11.33 (14.7%)	5.67 (19.5%)	1.33 (40.1%)
0.16:0.20:0.64	388.3 (7.2%)	91.00 (9.3%)	31.0 (11.7%)	7.67 (17.8%)	3.00 (27.7%)	1.00 (45.0%)
0.08:0.20:0.72	431.3 (6.8%)	95.67 (9.1%)	31.3 (11.6%)	9.67 (15.9%)	4.67 (21.3%)	1.33 (40.1%)
1.00:0.00:0.00	431.0 (6.8%)	106.3 (8.6%)	32.6 (11.4%)	10.00 (15.6%)	5.67 (19.5%)	1.00 (45.0%)
0.90:0.00:0.10	394.6 (7.2%)	102.6 (8.8%)	40.6 (10.1%)	12.33 (14.2%)	4.33 (22.5%)	2.33 (31.5%)
0.80:0.00:0.20	388.6 (7.2%)	85.00 (9.5%)	26.6 (12.6%)	9.00 (16.5%)	3.67 (24.5%)	1.67 (35.8%)
0.70:0.00:0.30	421.0 (6.9%)	90.33 (9.3%)	28.6 (12.3%)	9.00 (16.5%)	3.00 (27.7%)	1.00 (45.0%)
0.60:0.00:0.40	413.0 (7.0%)	105.0 (8.7%)	38.0 (10.4%)	17.00 (12.8%)	8.33 (16.8%)	2.67 (29.9%)
0.50:0.00:0.50	351.0 (7.6%)	92.67 (9.2%)	40.3 (10.2%)	15.67 (13.2%)	6.33 (18.6%)	2.33 (31.5%)
0.40:0.00:0.60	330.6 (7.9%)	79.67 (9.8%)	28.0 (12.4%)	11.67 (14.5%)	5.00 (20.8%)	0.33 (65.0%)
0.30:0.00:0.70	358.0 (7.6%)	82.00 (9.7%)	29.3 (12.1%)	12.67 (14.1%)	4.67 (21.3%)	3.33 (27.1%)
0.20:0.00:0.80	369.3 (7.4%)	89.67 (9.3%)	32.3 (11.5%)	13.00 (14.0%)	5.67 (19.5%)	1.67 (35.8%)

The hairiness profiles enable prediction of processing behavior and resultant fabric properties. Yarns with excessive long hairiness (≥ 4 mm > 15 hairs/10 m) may experience increased friction and potential challenges during subsequent weaving or knitting operations. High short hairiness contributes to fabric softness and fullness, while excessive long hairiness may increase pilling propensity^37,38^. Blends containing 0.20 grey with balanced chromatic components consistently show moderate hairiness across all length categories, suggesting optimal processing behavior.

When analyzing mechanical properties and surface characteristics together, strong statistical correlations emerge that confirm key structure–property relationships. A Pearson correlation analysis revealed a strong, statistically significant negative correlation between yarn evenness (U%) and tenacity (r = − 0.81, *p* < 0.001), providing quantitative proof for the fundamental principle that structural uniformity is critical for performance. This robust statistical link allows for the development of a quality-composition map that can predict multiple yarn properties based on blend ratios. The data identifies several optimal compositional ranges where multiple properties are simultaneously optimized. For example, the 0.40:0.20:0.40 blend not only showed one of the highest tenacity values but also maintained good evenness (U% 14.12% ± 0.78%), making its properties statistically superior to many other blends. These findings facilitate targeted design for specific end-use requirements while defining statistically validated processing windows where blend ratios consistently produce yarns meeting industry standards across multiple metrics.

**Fig. 9:**
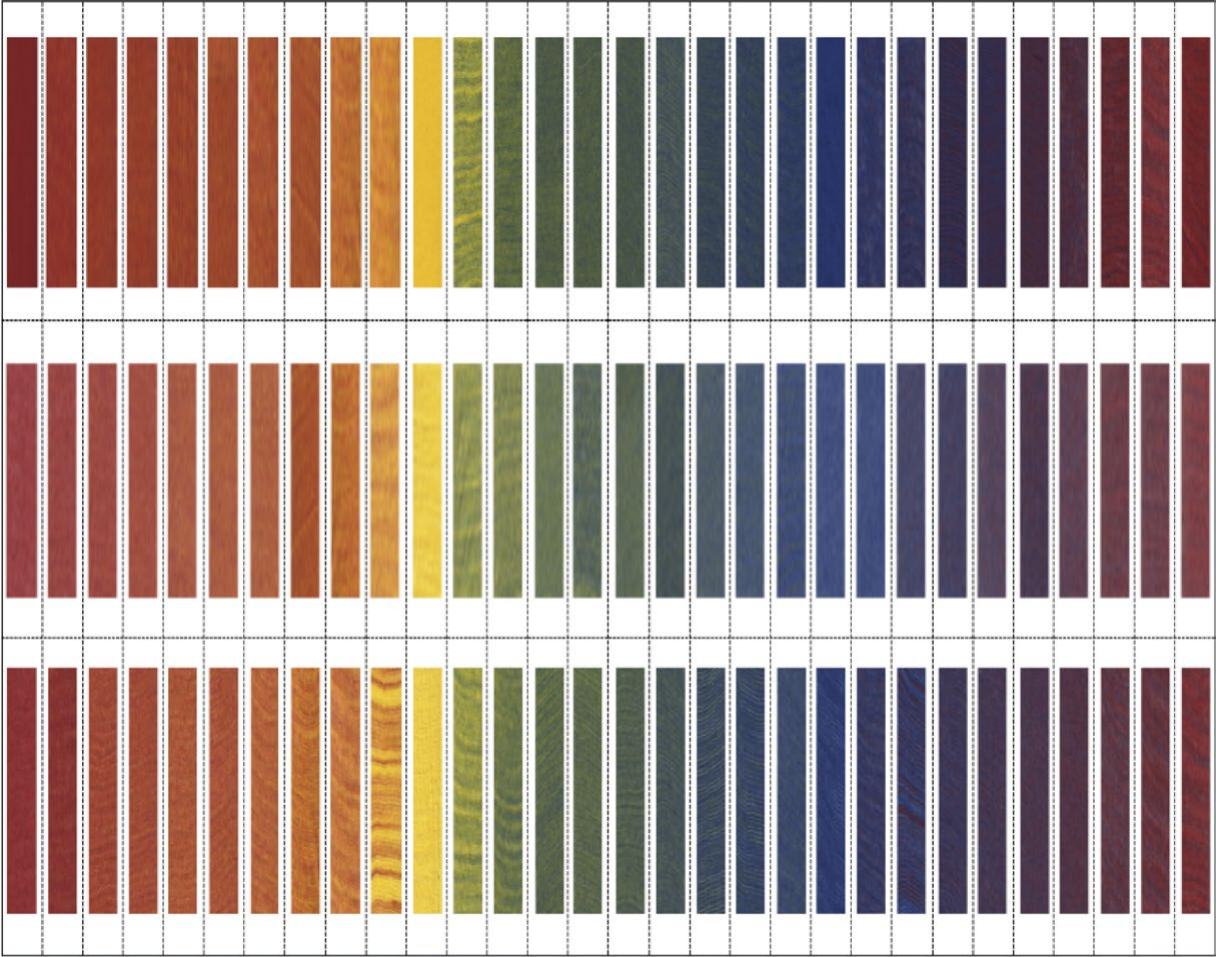
Scanned colors of the knitted fabric swatches.

Table 6 presents the comprehensive CIELAB color values for all 90 fabric samples measured using a 3nh YS6010 spectrophotometer under standardized conditions (D65 illuminant, 10° observer field). The visual representation of these measured values in Fig. 9 allows for direct comparison with our theoretical predictions from the panchromatic color model shown in Fig. 6. The remarkable correlation between theoretical predictions and experimental outcomes conclusively validates both the mathematical color prediction model and the efficacy of the digital control approach. Through precise manipulation of channel draft ratios, we successfully produced three distinct color series that systematically vary along key colorimetric dimensions. The consistent color reproduction achieved throughout our experiments demonstrates that the three-channel digital rotor spinning system, guided by our four-primary grid-based mixing model, provides unprecedented control over colorimetric parameters in color-spun yarn production. This enables textile designers and manufacturers to precisely target specific color coordinates without extensive trial sampling, representing a significant advancement in sustainable textile coloration technology.

**Table 6:** LAB color values of blended yarn.

No	l ; a ; b	No	l ; a ; b	No	l ; a ; b
C7,1	38.52,31.31,9.58	C9,1	33.69,9.78,129	C11,1	26.61,38.48,14
C7,2	41.49,29.27,11.9	C9,2	36, 32.97,13.7	C11,2	30.89,33.5,21.1
C7,3	41.56,28.69,17.1	C9,3	36.49,30.66,27.1	C11,3	34.92,31.74,24
C7,4	43.29,26.07,18.4	C9,4	40.48,26.08,23.8	C11,4	36.49,30.66,27
C7,5	47.08,22.73,24.4	C9,5	40.78,25.4,25.3	C11,5	37.54,27.47,28
C7,6	48.68,21.24,26	C9,6	44.8, 23.7,31.2	C11,6	40.36,26,32.9
C7,7	49.17,20.77,29.2	C9,7	44.02,24.44,27.5	C11,7	41.76,25.97,34
C7,8	52.72,18.80,34.8	C9,8	47.17,22.45,34.3	C11,8	43.01,24.7,35.7
C7,9	58 , 15.8 , 40.8	C9,9	54.92,15.22,43.4	C11,9	50.8,19.21,45.6
C7,10	62.59,11.47,48.1	C9,10	64.55, 10.7,54.4	C11,10	57.4,14.24,57
C7,11	77.83,5.04,72.3	C9,11	76.74, 4.61,71.9	C11,11	74.31,7.75,82
C7,12	60.84,− 8.85,36.8	C9,12	60.94, 6.58,50.4	C11,12	55.73,− 8.37,41.3
C7,13	54.01,− 9.6,23.4	C9,13	47.76, 8.83,21.8	C11,13	47.76,− 8.83,21.8
C7,14	46.71,− 9.89,8.36	C9,14	44.23, − 9.9,15.3	C11,14	42.63,− 10.4,18.7
C7,15	50.66,− 10.1,15.9	C9,15	43.69,− 11.2,13.6	C11,15	37.93,− 10.8,6.4
C7,16	44.8,− 9.91,7.61	C9,16	41.27,− 10.6,6.83	C11,16	40.61,− 11.6,14.1
C7,17	43.69,− 11.2,13.6	C9,17	37.53,− 7.83,− 3.7	C11,17	36.94,− 10.2,5.12
C7,18	42.82,− 5.95,− 7.2	C9,18	35.03,− 6.79,− 9.7	C11,18	32.78,− 7.55,8.90
C7,19	39.29,− 4.23,− 14	C9,19	34.68,− 4.27,− 18	C11,19	30.81,− 5.8,− 14
C7,20	40.86,− 2.41,− 19	C9,20	36.26,− 5.43,− 15	C11,20	29.03,− 3.03,− 21
C7,21	38.11,0.69,− 26	C9,21	32.76,− 0.29,− 29	C11,21	28.19,1.82,− 31
C7,22	37.93,0.76,− 26	C9,22	30.79,2.68,− 24	C11,22	26.48,3.93,− 27
C7,23	37.58,4.44,− 16	C9,23	32.07,1.45,− 29	C11,23	25.68,4.80,− 23
C7,24	34.75,5.33,− 17	C9,24	31.07,6.23,− 18	C11,24	24.92,8.45,− 17
C7,25	31.09,8.41,− 13	C9,25	30.52,8.67,− 13	C11,25	24.93,7.24,− 18
C7,26	36.69,7.29,− 13	C9,26	30.15,10.59,− 11	C11,26	24.06,13.07,− 10
C7,27	36.55,12.36,− 6.1	C9,27	29.79,14.08,− 7.8	C11,27	24.37,15.45,− 7.9
C7,28	36.78,15.71,− 3	C9,28	30.77,16.55,− 3	C11,28	24.73,25.55,2.5
C7,29	36.5,15.98,− 26	C9,29	29.78,22.14,0.97	C11,29	26.15,31.7,9.76
C7,30	38.12,21.5,1.29	C9,30	30.82,26.80,8.23	C11,30	26.69,33.44,9.7

## Conclusion

This research successfully developed and validated a digital control system for producing wide-gamut color-spun yarns. By integrating a four-primary grid-based color mixing model with a three-channel CNC rotor spinning system, a novel method was established that addresses key challenges in color-spun yarn production. Comprehensive experimental validation on 90 yarn samples confirmed that this approach enables the production of yarns with precise, predictable colors across the full gamut, while maintaining mechanical and evenness properties that meet or exceed industry standards. The excellent agreement between the predicted model and measured fabric colors demonstrates that the system effectively bridges the gap between theoretical color modeling and practical manufacturing, offering unprecedented precision. From a broader perspective, this research establishes a foundation for next-generation textile coloration technology that combines precision color control with sustainable manufacturing practices. By digitizing the color creation process, this approach can transform color-spun yarn production from a craft-based process to a precision-controlled digital technology.

However, to broaden the system’s applicability for industrial adoption, future work should address several critical factors. Firstly, the validation was conducted using a single 16G weft-knitted structure. It is well-established that fabric construction significantly influences final color appearance due to variations in yarn crimp, surface topography, and light-trapping effects. A key area for future research is therefore to quantify the "color transfer function" from the yarn to diverse fabric structures. Secondly, the experimental protocol was standardized to unfinished fabrics measured under a D65 illuminant. Industrial finishing processes (e.g., scouring, calendering) and the potential for metamerism under different illuminants necessitate the development of calibration models to account for these color shifts. Finally, the current model is validated only for 100% cotton. Since the model’s predictive accuracy depends on the material-specific input color matrix of the primary rovings, it must be recalibrated for other fiber types (e.g., polyester, wool, and blends) that possess distinct optical properties.

Furthermore, a detailed quantitative analysis of the color difference (ΔE) between the digital targets and the manufactured yarns would provide a more precise benchmark for the system’s reproduction fidelity and could be used to further refine the control algorithms.

## Supplementary Information

Below is the link to the electronic supplementary material.

Supplementary Material 1

## Data Availability

The datasets used and analyzed during the current study are available from the corresponding author upon reasonable request.
