# Supplementary material for: Digital control of wide gamut color spun yarns enabled by four primary grid based mixing model
Source: Sci Rep. 2025 Oct 15;15:36062. doi: 10.1038/s41598-025-19972-x (PMC12528671; doi:10.1038/s41598-025-19972-x)
Supplement: Supplementary file 1 — Supplementary Material 1 [file 41598_2025_19972_MOESM1_ESM.docx]

**Supplemental**

**Table S1**. Feed rates for three-channel rotor spinning (grey = 0.00)

| Blend ratio (blue: grey: red) | Blue feed rate (m/min) | Grey feed rate (m/min) | Red feed rate (m/min) |
| --- | --- | --- | --- |
| 1.00 0.00 0.00 | 2.00 | 0.00 | 0.00 |
| 0.90 0.00 0.10 | 1.80 | 0.00 | 0.20 |
| 0.80 0.00 0.20 | 1.60 | 0.00 | 0.40 |
| 0.70 0.00 0.30 | 1.40 | 0.00 | 0.60 |
| 0.60 0.00 0.40 | 1.20 | 0.00 | 0.80 |
| 0.50 0.00 0.50 | 1.00 | 0.00 | 1.00 |
| 0.40 0.00 0.60 | 0.80 | 0.00 | 1.20 |
| 0.30 0.00 0.70 | 0.60 | 0.00 | 1.40 |
| 0.20 0.00 0.80 | 0.40 | 0.00 | 1.60 |
| 0.10 0.00 0.90 | 0.20 | 0.00 | 1.80 |

**Table S2**. Feed rates for three-channel rotor spinning (Grey = 0.20)

| Blend ratio (blue: grey:red) | Blue feed rate (m/min) | Grey feed rate (m/min) | Red feed rate (m/min) |
| --- | --- | --- | --- |
| 0.80 0.20 0.00 | 1.60 | 0.40 | 0.00 |
| 0.72 0.20 0.08 | 1.44 | 0.40 | 0.16 |
| 0.64 0.20 0.16 | 1.28 | 0.40 | 0.32 |
| 0.56 0.20 0.24 | 1.12 | 0.40 | 0.48 |
| 0.48 0.20 0.32 | 0.96 | 0.40 | 0.64 |
| 0.40 0.20 0.40 | 0.80 | 0.40 | 0.80 |
| 0.32 0.20 0.48 | 0.64 | 0.40 | 0.96 |
| 0.24 0.20 0.56 | 0.48 | 0.40 | 1.12 |
| 0.16 0.20 0.64 | 0.32 | 0.40 | 1.28 |
| 0.08 0.20 0.72 | 0.16 | 0.40 | 1.44 |

**Table S3**. Feed rates for three-channel rotor spinning (Grey = 0.40)

| Blend ratio (blue: grey : red) | Blue feed rate (m/min) | Grey feed rate (m/min) | Red feed rate (m/min) |
| --- | --- | --- | --- |
| 0.60 0.40 0.00 | 1.20 | 0.80 | 0.00 |
| 0.54 040 0.06 | 1.08 | 0.80 | 0.12 |
| 0.48 0.40 0.12 | 0.96 | 0.80 | 0.24 |
| 0.42 0.40 0.18 | 0.84 | 0.80 | 0.36 |
| 0.36 0.40 0.24 | 0.72 | 0.80 | 0.48 |
| 0.30 0.40 0.30 | 0.60 | 0.80 | 0.60 |
| 0.24 0.40 0.36 | 0.48 | 0.80 | 0.72 |
| 0.18 0.40 0.42 | 0.36 | 0.80 | 0.84 |
| 0.12 0.40 0.48 | 0.24 | 0.80 | 0.96 |
| 0.06 0.40 0.54 | 0.12 | 0.80 | 1.08 |

**Table S4**. Feed rates for three-channel rotor spinning (grey = 0.00)

| Blend ratio (red: grey: yellow) | Red feed rate (m/min) | Grey feed rate (m/min) | Yellow feed rate (m/min) |
| --- | --- | --- | --- |
| 1.00 0.00 0.00 | 2.00 | 0.00 | 0.00 |
| 0.90 0.00 0.10 | 1.80 | 0.00 | 0.20 |
| 0.80 0.00 0.20 | 1.60 | 0.00 | 0.40 |
| 0.70 0.00 0.30 | 1.40 | 0.00 | 0.60 |
| 0.60 0.00 0.40 | 1.20 | 0.00 | 0.80 |
| 0.50 0.00 0.50 | 1.00 | 0.00 | 1.00 |
| 0.40 0.00 0.60 | 0.80 | 0.00 | 1.20 |
| 0.30 0.00 0.70 | 0.60 | 0.00 | 1.40 |
| 0.20 0.00 0.80 | 0.40 | 0.00 | 1.60 |
| 0.10 0.00 0.90 | 0.20 | 0.00 | 1.80 |

**Table S5**. Feed rates for three-channel rotor spinning (grey = 0.20)

| Blend ratio (red: grey: yellow) | Red feed rate (m/min) | Grey feed rate (m/min) | Yellow feed rate (m/min) |
| --- | --- | --- | --- |
| 0.80 0.20 0.00 | 1.60 | 0.40 | 0.00 |
| 0.72 0.20 0.08 | 1.44 | 0.40 | 0.16 |
| 0.64 0.20 0.16 | 1.28 | 0.40 | 0.32 |
| 0.56 0.20 0.24 | 1.12 | 0.40 | 0.48 |
| 0.48 0.20 0.32 | 0.96 | 0.40 | 0.64 |
| 0.40 0.20 0.40 | 0.80 | 0.40 | 0.80 |
| 0.32 0.20 0.48 | 0.64 | 0.40 | 0.96 |
| 0.24 0.20 0.56 | 0.48 | 0.40 | 1.12 |
| 0.16 0.20 0.64 | 0.32 | 0.40 | 1.28 |
| 0.08 0.20 0.72 | 0.16 | 0.40 | 1.44 |

**Table S6**. Feed rates for three-channel rotor spinning (Grey = 0.40)

| Blend ratio (red: grey: yellow) | Red feed rate (m/min) | Grey feed rate (m/min) | Yellow feed rate (m/min) |
| --- | --- | --- | --- |
| 0.60 0.40 0.00 | 1.20 | 0.80 | 0.00 |
| 0.54 040 0.06 | 1.08 | 0.80 | 0.12 |
| 0.48 0.40 0.12 | 0.96 | 0.80 | 0.24 |
| 0.42 0.40 0.18 | 0.84 | 0.80 | 0.36 |
| 0.36 0.40 0.24 | 0.72 | 0.80 | 0.48 |
| 0.30 0.40 0.30 | 0.60 | 0.80 | 0.60 |
| 0.24 0.40 0.36 | 0.48 | 0.80 | 0.72 |
| 0.18 0.40 0.42 | 0.36 | 0.80 | 0.84 |
| 0.12 0.40 0.48 | 0.24 | 0.80 | 0.96 |
| 0.06 0.40 0.54 | 0.12 | 0.80 | 1.08 |

**Table S7**. Feed rates for three-channel rotor spinning (Grey = 0.00)

| Blend ratio (yellow: grey: blue) | Yellow feed rate (m/min) | Grey feed rate (m/min) | Blue feed rate (m/min) |
| --- | --- | --- | --- |
| 1.00 0.00 0.00 | 2.00 | 0.00 | 0.00 |
| 0.90 0.00 0.10 | 1.80 | 0.00 | 0.20 |
| 0.80 0.00 0.20 | 1.60 | 0.00 | 0.40 |
| 0.70 0.00 0.30 | 1.40 | 0.00 | 0.60 |
| 0.60 0.00 0.40 | 1.20 | 0.00 | 0.80 |
| 0.50 0.00 0.50 | 1.00 | 0.00 | 1.00 |
| 0.40 0.00 0.60 | 0.80 | 0.00 | 1.20 |
| 0.30 0.00 0.70 | 0.60 | 0.00 | 1.40 |
| 0.20 0.00 0.80 | 0.40 | 0.00 | 1.60 |
| 0.10 0.00 0.90 | 0.20 | 0.00 | 1.80 |

**Table S8**. Feed rates for three-channel rotor spinning (grey = 0.20)

| Blend ratio (yellow: grey: blue) | Yellow feed rate (m/min) | Grey feed rate (m/min) | Blue feed rate (m/min) |
| --- | --- | --- | --- |
| 0.80 0.20 0.00 | 1.60 | 0.40 | 0.00 |
| 0.72 0.20 0.08 | 1.44 | 0.40 | 0.16 |
| 0.64 0.20 0.16 | 1.28 | 0.40 | 0.32 |
| 0.56 0.20 0.24 | 1.12 | 0.40 | 0.48 |
| 0.48 0.20 0.32 | 0.96 | 0.40 | 0.64 |
| 0.40 0.20 0.40 | 0.80 | 0.40 | 0.80 |
| 0.32 0.20 0.48 | 0.64 | 0.40 | 0.96 |
| 0.24 0.20 0.56 | 0.48 | 0.40 | 1.12 |
| 0.16 0.20 0.64 | 0.32 | 0.40 | 1.28 |
| 0.08 0.20 0.72 | 0.16 | 0.40 | 1.44 |

**Table S9**. Feed rates for three-channel rotor spinning (grey = 0.40)

| Blend ratio (yellow: grey: blue) | Yellow feed rate (m/min) | Grey feed rate (m/min) | Blue feed rate (m/min) |
| --- | --- | --- | --- |
| 0.60 0.40 0.00 | 1.20 | 0.80 | 0.00 |
| 0.54 040 0.06 | 1.08 | 0.80 | 0.12 |
| 0.48 0.40 0.12 | 0.96 | 0.80 | 0.24 |
| 0.42 0.40 0.18 | 0.84 | 0.80 | 0.36 |
| 0.36 0.40 0.24 | 0.72 | 0.80 | 0.48 |
| 0.30 0.40 0.30 | 0.60 | 0.80 | 0.60 |
| 0.24 0.40 0.36 | 0.48 | 0.80 | 0.72 |
| 0.18 0.40 0.42 | 0.36 | 0.80 | 0.84 |
| 0.12 0.40 0.48 | 0.24 | 0.80 | 0.96 |
| 0.06 0.40 0.54 | 0.12 | 0.80 | 1.08 |
